# High-throughput transcriptome sequencing of the cold seep mussel *Bathymodiolus platifrons*

**DOI:** 10.1038/srep16597

**Published:** 2015-11-23

**Authors:** Yue Him Wong, Jin Sun, Li Sheng He, Lian Guo Chen, Jian-Wen Qiu, Pei-Yuan Qian

**Affiliations:** 1 Division of Life Science, School of Science, the Hong Kong University of Science and Technology, Hong Kong S.A.R; 2 Department of Biology, Hong Kong Baptist University, Hong Kong S.A.R; 3 Sanya Institute of Deep-sea Science and Engineering, Chinese Academy of Sciences, Hainan, People Republic of China

## Abstract

Bathymodiolid mussels dominate hydrothermal vents, cold methane/sulfide-hydrocarbon seeps, and other sites of organic enrichment. Here, we aimed to explore the innate immune system and detoxification mechanism of the deep sea mussel *Bathymodiolus platifrons* collected from a methane seep in the South China Sea. We sequenced the transcriptome of the mussels’ gill, foot and mantle tissues and generated a transcriptomic database containing 96,683 transcript sequences. Based on GO and KEGG annotations, we reported transcripts that were related to the innate immune system, heavy metal detoxification and sulfide metabolic genes. Our in-depth analysis on the isoforms of peptidoglycan recognition protein (PGRP) that have different cellular location and potentially differential selectivity towards peptidoglycan (PGN) from gram-positive and gram-negative bacteria were differentially expressed in different tissues. We also reported a potentially novel form of metallothionein and the production of phytochelatin in *B. platifrons*, which has not been reported in any of its coastal relative *Mytilus* mussel species. Overall, the present study provided new insights into heavy metal and sulfide metabolism in *B. platifrons* and can be served as the basis for future molecular studies on host-symbiont interactions in cold seep mussels.

In deep sea hydrothermal vent and hydrocarbon seep ecosystems, chemosynthetic microbes are the primary producers^1^. Symbiosis between vent or seep macro-faunal such as mussels, snails, shrimp, tubeworms and crabs and chemosynthetic microbes is a common adaptive mechanism^2^,^3^,^4^. In these symbiotic systems, the microbial biomass serves as either the major or the sole food source of the host^5^. Vent and seep effluents are also known to enrich in heavy metal, sulfide and different hydrocarbon species^6^,^7^. Hence, in addition to acquisition of chemosynthetic microbes as symbionts, both vent and seep macro-fauna have to adapt to a highly toxic chemical environment. Among different deep sea macro-fauna, Bathymodiolid mussels represent one of the highly specialized animals to vent and seep ecosystems. These deep sea mussels are evolved with the mechanisms to acquire special nutritional advantage from chemosynthetic bacteria and to tolerate a range of highly toxic chemicals. These adaptive features have enabled Bathymodiolid mussels to flourish and dominate hydrothermal vents, cold methane/sulfide-hydrocarbon seeps, and other sites of organic enrichment (e.g., sunken wood and whale bones) in the Atlantic, Pacific, and Indian Oceans^8^.

Bathymodiolid mussels are capable of acquiring chemoautotrophic bacteria as their major nutritional food source^9^. Instead of a vertical transmission of microbial symbionts, Bathymodiolid mussels actively acquire thiotrophic and/or methanotrophic gamma-proteobacteria starting from juvenile stage^10^. These bacterial symbionts are maintained in bacteriocytes, a type of hemocytes with specialized cellular compartment for the storage of symbiotic bacteria^10^. Bacteriocytes may be absorbed via phagocytosis occasionally for nutritional purposes^10^. To date, it remains unclear how Bathymodiolid *mussels* distinguish pathogens from symbionts and how symbionts avoid triggering adverse immune responses from the host.

Bathymodiolid mussels have also been reported to accumulate high concentration of heavy metals, and tolerate high levels of toxic hydrocarbons and sulfide^11^,^12^. However, majority of the studies concerning heavy metal accumulation and sulfide detoxification focused on vent mussels. For instance, Metallothionein genes from several vent dwelling *Bathymodiolus* species have been reported^13^. A recent high-throughput sequencing analysis of the vent mussel *Bathymodiolus azoricus* has revealed an extensive collection of innate immune transcripts in the gill^14^. However, the metabolism potential of the vent mussel in term of heavy metal and sulfide detoxification were not explored.

The objective of this study was to generate a comprehensive transcriptome database for the methane seep mussel *Bathymodiolus platifrons* (see Fig. 1). To gain an insight into the adaptive features of the seep mussel, we focused on genes related to immune function and detoxification. We perform in-depth analysis on these functional genes and analyzed their gene expression pattern in the gill, mantle and foot. This work extended our understanding on the mechanisms of detoxification and introduced new perspective on the mechanism of symbiont acquisition in Bathymodiolid mussels.

## Results

### *De novo* assembly and functional annotation of *B. platifrons* transcriptome

The mitochondrial COI and NADH4 gene sequences of the specimen had highest similarity (99% and 100%, respectively) to the respective *B. platifrons* sequences from Okinawa Trough, Hatoma Knoll^15^. We produced five Gbp clean data (approximately 55 million clean reads) from each of the three tissues (Table S1). Over 98% clean Illumina reads in all three tissues exceeded Q20, indicating high quality of the sequencing data. The raw sequencing data have been submitted to the Short Read Archive (SRA) of NCBI under accession number SRP035485. The final transcriptome generated from Trinity *de novo* assembling contained 96,683 transcript sequences. The length of the transcripts ranged from 200 to 24,272 bp, with an average length of 673 bp and N50 value of 983 bp. The statistics for the data output and *de novo* assemblies are summarized in Table 1. The length distribution of all transcripts is shown in Figure S1. In total, 40,935 (42.3%) transcripts had at least one significant hit in the NCBI non-redundant (nr), Swiss-Prot, COG and KEGG protein database. The functional annotation results are shown in Table 1. The COG and KEGG functional categories for the annotated transcripts are shown in Fig. 2A,B, respectively. There were 14,656 (39.5%) nr-annotated transcripts assigned to major Gene Ontology (GO) categories, i.e. ‘Biological Process’, ‘Cellular Component’, and ‘Molecular Function’ (Fig. 2C). ESTscan and BLAST search of all the aforementioned protein databases resulted in the prediction of 44,633 protein coding transcripts (Table 1).

To evaluate the comprehensiveness of the current *B. platifrons* transcriptome assembly, we downloaded 458 single-copy genes (the core proteins) from the Core Eukaryotic Gene Mapping Approach (CEGMA) dataset^16^ and using tBLASTn with a evalue cut-off of 10^−3^ to assay the completeness of our transcriptome assembly. All the 458 core proteins have at least one match in the current *B. platifrons* transcriptome (Supplemental data file 1). The average percentage of identity of alignment was 70% (S.D. 12.7%). This result indicated the high quality and the comprehensiveness of our assembled transcriptome.

The best hit of the majority of the annotated transcripts (15,005 out of 37,039) are *Crassostrea gigas* sequences in the nr database. This is reasonable as *C. gigas* is thus far the only bivalve whose genome has been sequenced (Fig. 2D). There were 1,402 bacterial transcripts (Fig. 2D, indicated by an asterisk). Approximately 0.2%, 0.09% and 0.12% of the clean Illumina reads from the gill, foot and mantle, respectively, were mapped to these bacterial transcripts. The LCA assignment algorithm assigned 225 bacterial transcripts to Methylococcaceae and 367 to Bradyrhizobiaceae (Fig. 2E). In general, transcripts assigned to Methylococcaceae were highly expressed in the gill while those assigned to Bradyrhizobiaceae were highly expressed in the foot (Supplemental data file 2). Among those 225 bacterial transcripts to Methylococcaceae, metabolic functional genes that play essential role in methane oxidation, sulfur reduction and nitrite/nitrate reduction were recovered (Fig. S2).

### Function of dominant transcripts

Close to half of the dominant transcripts (top 5% transcripts in term of total expression level) have no match to proteins in any public database (Fig. S3B). The ten most enriched KEGG pathways and GO Biological Process (GOBP) terms among the dominant transcript list are shown in Fig. 3C,D, respectively. The full list of enriched KEGG pathways and GOBP terms are shown in Table S2 and S3 respectively. In both KO and GOBP enrichment analyses, terms related to *de novo* synthesis (KEGG pathways: “splicesosome”, “RNA transport” and “ribosome”; GOBP term: “translation”) were highly enriched in the dominant transcript list (Fig. S3C & S3D). Oxidative phosphorylation” pathway was the most enriched KEGG pathway in the dominant transcript list (Fig. S3C), indicating a high level of energy requirement and active engagement of the mussel in *de novo* synthesis.

### Differential expression analysis

We then compared the gene expression profiles of the three examined tissues to identify differentially expressed genes. As quantification accuracy is positively correlated with sequence coverage^17^, only 7,304 transcripts that have at least 750 clean reads (equivalent to 5 ppm total mapped reads) mapped to all three examined tissues in summation were considered in the differential expression analysis. In each pairwise comparison, differentially expressed transcripts was defined as log2(ratio) values greater than 2 SD from the mean. The mean, SD and cutoff log2(ratio) values for differentially expressed transcripts in each pairwise comparison were listed in Table 2. We focused on transcripts that shown distinctively higher expression level in one of the examined tissues when compared with the other two. We found that transcripts that were dominantly expressed in gill [transcripts highly expressed in the gill in both gill-to-mantle (G-to-M) and gill-to-foot (G-to-F) comparisons] are enriched with genes involved in immune system (*GO:0002376-immune system process*) (see Fig. 4, the GO term is highlighted in red color). Both foot (transcripts highly expressed in the foot in G-to-F and foot-to-mantle (F-to-M) comparisons) and mantle dominant transcripts (transcripts highly expressed in the mantle in both G-to-M and F-to-M comparisons) were enriched with genes with developmental functions/regulations (Fig. 4). In accord to the biomineralization function of mantle in bivalves^18^, the term *ion transport* (GO:0006811) was found to be enriched in the mantle dominant transcript list. More detail analyses on mantle dominant transcripts were reported in Sun *et al.* (Submitted).

As we were aiming at generating a database for *B. platifrons*, we did not have replications on our RNAseq data. Hence, we verified the tissue dominant pattern of gene expression detected from RNAseq using qRT-PCR. Different from the RNAseq data, the qRT-PCR experiments were conducted with 3 biological replicates (i.e. 3 individuals) and 2 experimental repeats per biological replicate. We randomly selected 10 transcripts for qRT-PCR quantification from each of the above tissue dominant transcript list. In general, the qRT-PCR results were in good agreement to the result of RNAseq quantification, as indicated by positive linear correlations and rather high Pearson product-moment correlation coefficients (R^2^ for Gill-to-Foot: 0.9582; R^2^ for Gill-to-Mantle: 0.9697; R^2^ for Foot-to-Mantle: 0.8532; see Fig. S3). This result indicated the high reliability of our RNAseq-based comparison of tissue dominant gene expression patterns. Annotation information and the relative expression level as calculated from RNAseq and qRT-PCR results were provided in Table S4.

### Phytochelatin synthase and dominant expression of metallothioneins

Phytochelatin, the oligomer of glutathione is one of the major molecules directly involved in metal ion detoxification^19^. We detected one transcript (Unigene479_All) best matched to glutathione gamma-glutamylcysteinyltransferase 3, or phytochelatin synthase (PSC) from the Pacific Oyster *Crassostrea gigas*. Unigene479_All did not show substantial variation in the three examined tissue. Alignment of *B. platifrons* PCS protein sequence with the orthologs from mollusks (*C. gigas*, *Aplysia californica*), urochordate (*Ciona intestinalis*), nematode (*C. elegans*), and plant (*Arabidopsis thaliana*) revealed that the N-terminal PC synthase domain in *B. platifrons* PCS (pfam domain no. PF05023, HMM prediction evalue 4.1^e−79^) is highly conserved, but the C-terminal region is highly variable (see Fig. 4C). This result is consistent with the alignment result of PCSs from plant, yeast and nematode^20^. Searching through the current transcriptome assembly, we found that the enzymes involved in major reactions in biosynthesis of phytochelatin were detected but did not show drastic expressional variation (not exceeding a two-fold difference) in the three examine tissues (see Fig. 4A,B). Nonetheless, our data suggested that the mussel is able to produce PC in all examined tissues. PCS sequences were also recovered in *B. azoricus* transcriptome (Fig. S5), as revealed by Blastp search of Unigene479 against the DeepSeaVent database, the customized protein database derived from *B. azoricus* gill transcriptome^21^. *B. platifrons* PCS clustered with PCS from *C. gigas* and *Aplysia californica* in the phylogenetic analysis (Fig. 4D), suggesting that the PCS in both *Bathymodiolus* mussel transcriptomes are unlikely to be originated from parasitic organisms.

We then examined metallothionein (MT), a well-studied metal chelating protein. All three MT transcripts (Unigene42054, CL8066.Contig1, CL8066.Contig2) were predominantly expressed in all the three examined *B. platifrons* tissues (Table S5). Former studies on the MT from *B. azoricus* and *B. thermophiles* reported two MT variants, MT-10 and MT-20^17^. Our phylogenetic analysis suggested that, while Unigene42054 is homologous to the MT-20 cluster, CL8066.Contig1 and CL8066.Contig2 are clearly non-homologous to either *Mytilidae* or vertebrate MTs (Fig. 5A). In fact, while CL8066.Contig1_All were poorly aligned to *Daphnia pulex* MT (bitscore 33.5, Evalue 1.8), CL8066.Contig2_All has no significant match in nr (Table S5). However, the metallothionein-like proteins encoded by these two transcripts contain seven Cys-Xaa-Cys motifs (indicated by pale red boxes in Fig. 5B), suggesting their potential role in metal ion binding. On the other hand, *B. platifrons* MT-20 (product of Unigene42054) contains nine of Cys-Xaa-Cys motifs (indicated by pale red boxes in Fig. 5B) that are conserved within Mytilidae. Comparing to the MT-20 from *B. thermophilus* and *B. azoricus, however, B. platifrons* MT-20 as well as *M. edilus* MT-20 has one less Cys-Xaa-Cys motif at the 55^th^–57^th^ residue (indicated by pale green box in Fig. 5B).

### Sulfur metabolism

We detected four transcripts of sulfite reductase (three alpha and one beta component), which catalyzes the oxidation of sulfide to sulfite, and two of sulfite oxidase, which catalyzes the oxidation of sulfite to sulfate, in the current *B. platifrons* transcriptome. Both sulfite oxidase transcripts were predominantly expressed in gill (Table 2). Based on the nr annotation, three of the sulfite reductase transcripts (Unigene8776_All, Unigene19303_All and Unigene58781_All) were from eukaryotic source while the remaining one transcripts (Unigene46612_All) was from methane-oxidizing bacteria. We also detected three transcripts annotated as sulfide:quinone oxidoreductase (SQR), a membrane bound mitochondrial protein catalyzing the formation of polysulfide from sulfide using a quinone molecule^22^. Two of the SQR transcripts shown a relative higher expression in gill, followed by foot and then mantle. The transcripts of taurine transporter (TAUT), which was implicated in sulfide detoxification and supplying sulfur to thiotrophic symbionts in Bathymodiolid mussels^23^,^24^, exhibited higher expression in the mantle rather than in the gill.

### Expression patterns of genes implicated in immune response

We found a repertoire of immune system genes that were known to be up-regulated in response to experimental introduction of virulent factors such as peptidoglycans, lipopolysaccharides and beta-glucans, in *C. gigas* or coastal mussels *Mytilus* spp. (Table S6). The list included pattern recognizing proteins peptidoglycan recognition protein (PGRP), Toll-like receptor and bactericidal permeability increasing protein (BPI), downstream transcription factor Lipopolysaccharide-induced tumor necrosis factor-alpha factor (LITAF), inflammatory cytokines interleukin-17 (IL-17) and tumor necrosis factor (TNF), antimicrobial peptides big defensing (BD) and macin. Certain transcripts of PGRP, BPI, TLR, IL-17, BD and macin exhibited distinctive tissue expression pattern (at least three fold differences) in either one of the three pairwise comparisons (gill-to-foot, gill-to-mantle, foot-to-mantle)) (highlighted by colored box in Table S6). To further investigate the potential molecular mechanisms of the host-symbiont interaction, in the following paragraphs, we focused on the PGRP, one of the crucial microbial associated molecule pattern recognition proteins, for sequence homology analysis.

### Differential expression of peptidoglycan recognition protein isoforms

In the current *B. platifrons* transcriptome, 11 transcripts were matched to PGRP sequences (Table S6). Two of the 11 transcripts were grouped under the cluster CL8290 and eight under CL2060. All the PGRPs encoded by the transcripts in CL2060 were predicted to contain an N-terminal signal peptide, followed by a propeptide and then by the amidase domain (Fig. 6A). This result indicated the differences in cellular localization of different PGRPs isoforms. It is unknown at this stage if the PGPR encoded by Unigene3808 is secretory or not as Unigene3808 is a 3′ partial sequence. Based on sequence homology, however, this transcript was likely to be derived from the same gene as the contigs from CL2060 (Fig. 6A). Multiple sequence alignment with insect and vertebrate PGRPs (see Fig. S8) revealed that all the amino acid residues essential for zinc binding (black arrow in Fig. 6A)^25^ and amidase activity (purple arrow in Fig. 6A)^26^ were conserved in all the 11 PGRP transcripts. Several insect based studies revealed two variable residues (indicated by red asterisk in Fig. 6A,B) that might affect peptidoglycan (PGN) binding selectivity towards Lysine-type (Lys-type) and diaminopimelic acid-type (Dap-type) PGNs^27^,^28^,^29^,^30^,^31^,^32^. We found that these two transcript variants also exist among *B. platifrons* PGRPs. For the PGRPs encoded by transcripts from CL8290 and CL2060, the two residues corresponding to the two variable residues in insect PGRPs were Gly/Arg-Trp. For PGRP encoded by Unigene3808_All, the two corresponding residues were Gln-Phe. These suggested that PGRPs encoded by transcripts from CL8290 and CL2060 might favor the binding of the Dap-type PGNs^28^ while that from Unigene3808_All might favor binding of Lys-type PGNs (indicated by red asterisk in Fig. 6B)^30^. Differential expression analysis based on our RNAseq data revealed that Unigene3808 had highest expression level in the foot while transcripts from CL8290 and CL2060 shown higher expression in the gill and mantle. Such differential expression pattern of these PGRP transcripts were confirmed by qRT-PCR assays (Fig. 6C) using primers specifically recognizing PGRP isoforms with Gly-Trp, Arg-Trp or Gln-Phe residues (Fig. 6B). Taken together, our results indicate that isoforms of PGRP may have differential selectivity towards different types of PGNs, and with different abundance in different tissues.

## Discussion

Our primary objective was to produce a comprehensive transcriptome database for each examined tissue for the methane seep dwelling *B. platifrons*. Hence, we pooled the total RNA of each tissue from three individuals during the cDNA library preparation step to maximize the sequencing coverage with the same amount of output. Because there were no replicates, our differential gene expression comparisons among the three tissues only targeted genes that were remarkably different. As quantification accuracy is positively correlated with sequence coverage^17^, transcripts with low coverage (with less than 5 ppm total mapped reads) were filtered out. Similar filtering approach (10 ppm total mapped reads as cutoff) was adopted in a coral transcriptomic study^33^. Our qRT-PCR assays results of 30 differential expressed transcripts shown good agreement with the RNAseq results, indicating that our approach on differential tissue expression analysis was valid.

In the current study, we unexpectedly recovered 1,402 microbial transcripts in our *B. platifrons* transcriptome assembly, in which up to 16% of these transcripts were likely to be originated from the methanotroph symbiont. Bacterial genes from the bacterial family Methylococcaceae were also detected in a transcriptomic study of *B. azoricus*^14^. In prokaryotes, mRNA is mature upon transcription^34^ and only a small fraction of mRNAs is polyadenylated at any given time^35^. Instead of stabilizing the RNA molecules, polyadenylation of RNA promotes degradation^36^. Hence, the microbial transcripts in *B. platifrons* transcriptome may represent a fraction of predominant bacterial mRNA that have high turnover rate. While the discovery of transcripts related to methane oxidation was not surprising, the gill specific expression of denitrification genes was unexpected. This may indicate that the methanotrophic endosymbionts might be actively catalyzing the production of ammonium via denitrification. A previous study has reported Bathymodiolid mussels to actively assimilate ammonium and free amino acids but not nitrate^37^. The authors of that study estimated that, in the presence of methane, the acquisition rate of ammonium correlated with the carbon fixation rate^37^. Our transcriptomic data suggested that, in addition to acquiring host ammonia waste, the methanotrophic symbiont may also be able to produce ammonia as their nitrogen source. On the other hand, substantial number of foot dominant bacterial transcripts was assigned to the nitrogen-fixing bacterial family Bradyrhizobiaceae. To date, no reports have described endo- or ectosymbiosis in the foot of any *Bathymodiolus* mussel. Dang *et al.*^38^ recovered diverse nifH and nifH-like gene sequences from the surface sediments of the methane seep in the Okhotsk Sea In addition, Rhizobia have been isolated from organic rich sapropel layers in the Mediterranean seabed^39^. These studies suggested the presence of nitrogen-fixing bacteria in the sediment of the methane seep and these nitrogen-fixing bacteria might be attached to the surface of the foot tissue of the mussel during sediment digging. Nonetheless, the presence of Bradyrhizobiaceae bacteria in the foot has to be validated by 16 s amplicon pyrosequencing.

In the present study, we found that a number of pattern recognition proteins such as PGRP, BPI, Toll-like receptors were highly expressed in the gill compared to the foot and mantle. Yet, our RNAseq data showed that different isoforms of the same gene the expression of immune system genes can exhibit highly variable expression pattern. For instance, while all *B. platifrons* PGRP isoforms contain amidase domain, they may have different binding affinity toward Gram-positive bacterial (Lys-type) and Gram-negative and Gram-positive bacilli (Dap-type) PGNs. Our RNAseq data indicated that the Dap-type PGNs binding PGRPs have a much higher expression level in the gill compared to the foot. The Lys-type PGNs binding PGRP, on the other hand, exhibited a slightly higher expression level in the foot compared to the gill. It is worth noting that the selectivity of PGRPs to Lys-type and Dap-type PGNs may not be absolute^26^,^32^. At this stage, we cannot confirm if different PGRPs isoforms in *B. platifrons* really exhibit selectivity toward different types of PGN. Nevertheless, we speculate that the expression patterns of different PGRP isoforms were related to the gram-negative methanotrophic symtbiont in the gill of *B. platifrons*. In insects, the PGRP-LB has been shown to be a key factor in suppressing virulent bacteria. It has been suggested that the outermembrane protein OmpA of the endosymbiotic bacteria *Sodalis glossinidus* can interact with the Tse-Tse fly PGRP-LB (one of the long PGRP splice variants). Binding of OmpA to PGRP-LB lead to suppression of the immunodeficiency (Imd) pathway^40^ and thereby the insect host immune response. Given that 1) PGRP-LB is required in insect-symbiont interaction, 2) the C-terminal sequence of major outer membrane protein (MopB) of the methanotrophic bacteria *Methylococcus capsulatus* shows significant identity to the OmpA family^41^, and 3) these PGRPs were dominantly expressed in the gill, we propose that *B. platifrons* PGRPs is a good candidate gene for studying the mechanism of immunotolerance of the *Bathymodiolus* mussel host to the methane-oxidizing bacteria.

The endemism of *B. platiftrons* in both hydrothermal vent and methane seeps in the Pacific Ocean^8^ suggested that this Bathymodiolids species has the metabolic capacity to metabolize a range of toxic chemical substances in these habitats. Distinctively high heavy metal concentration is one of the signposts of hydrothermal vent and methane seep habitats^6^,^7^. High concentration of heavy metal ions promotes the production of reactive oxygen species, which could be hazardous to cellular metabolic reactions^42^. Hemostasis of heavy metal ions is therefore of crucial importance in maintaining a stable intracellular chemical environment. Up-regulation of MTs, a class of ubiquitously expressing low molecular weight cysteine-rich metal ion binding proteins implicated in heavy metal homeostasis^42^, has been documented in various bivalves after exposure to heavy metals^43^,^44^,^45^. The dominant expression of MT in *B. platifrons* indicated high heavy metal level in the seep environment. A group of MT-like proteins, which contains seven Cys-Xaa-Cys motifs but did not show any conservation (except the Cys-Xaa-Cys motifs) to any known MTs, was recovered from the current *B. platifrons* transcriptome. Further *in vitro* experiments are needed to determine the affinity of this MT-like protein toward different metal ions.

One of the surprising finding in term of heavy metal detoxification was the discovery of glutathione (GSH) gamma-glutamylcysteinyltransferase, or phytochelatin synthase (PCS). Phytochelatin(PC) contains repeats of υ-GluCys dipeptide followed by a terminal Gly. In the presence of metal ion, PCS catalyzed the transpeptidation of the υ-GluCys moiety of GSH onto a second GSH molecule to form repeats of PC, or a υ-GluCys dipeptide-metal complex^20^,^46^. *In vivo* study using PC from plants demonstrated that PC preferably chelates Cd, but can also form complex with Ag and Cu^47^. PCS has been mainly characterized in plant, yeast and parasitic nematodes^47^,^48^,^49^. Hence, we did suspect that the recovery of PCS in *B. platifrons* transcriptome might have been due to the contamination by small parasitic organisms such as nematodes. These small organisms were hard to be seen and removed during sampling. Recently, however, PCS has been reported in several molluscan species, including the Pacific Oyster *C. gigas* genome^50^. More importantly, sequence alignment as well as phylogenetic analysis suggested that the PCS in *B. platifrons* transcriptome clustered with the PCS from *C. gigas*. In fact, PCS was also expressed in the gill of *B. azoricus* (as indicated by the result of reciprocal blast search, Fig. S6). This suggested that *B. azoricus* also has the potential to produce PC. This result further supported that the PCS sequence in *B. platifrons* transcriptome was not from a contamination source. There is so far no report of PCS gene or production of PC in any member of Mytilidae. This may imply that either PCS gene is specific to Bathymodiolid mussels among Mytilidae or previous high-throughput studies on coastal member of Mytilidae (such as *Mytilus* spps.) have missed out PCS gene expression, owing to limitations on sequencing or annotation. Detection of PC in *B. platifrons* tissues by analytical chemistry method is required to validate if the mussel is really producing PC *in vivo*. It will also be essential to examine if PC biosynthesis increase in response to heavy metal administration.

Hydrogen sulfide is another major component in vent and seep effluent^8^,^9^. The compound is highly inhibitive to aerobic respiration. While the blood of tubeworm and another bivalve were capable of buffering the acidifying effect of hydrogen sulfide^51^,^52^, the blood of Bathymodiolid mussels did not show such buffering characteristics, implying that there is no sulfide-binding protein in its blood^53^. Instead, it was suggested that sulfide oxidase activity in both the symbiont-containing gill tissue and peripheral lining of other symbiont-free tissues serves as “peripheral defense” line to oxidize any sulfide that enters the cells to sulfite^53^. In the current study, we found that, except sulfite oxidase, the expression of mitochondrial enzymes that participate in sulfide oxidation or sulfide detoxification did not show drastic differential expression among the three examined tissues. This finding is in accordance with the existence of a peripheral defense line against sulfide in both symbiont-containing and symbiont-free tissues. Comparing to sulfite reductase, transcripts of SQR showed a much higher expression value (rpkm). SQR catalyzes the formation of polysulfide from sulfide. SQR in lugworms and other marine invertebrates that inhabit high sulfide environments^54^ avoid elevation of intracellular sulfide concentrations. Abundant expression of SQR in *B. platifrons* suggested that the mussel may mainly remove sulfide by the mean of SQR catalyzing polysulfide formation.

High levels of thiotaurine has been found in the tissues of vent and seep invertebrates, leading to the hypothesis that the unusual amino acid is involved in the detoxification of sulfide^55^,^56^,^57^. In the thiotrophic symbiont harboring *B. septemdierum*, the expression level of the taurine transporter TAUT was higher in the gill compared to the foot and mantle^58^. This result has led Kioto *et al.*^58^ to propose that the mussel gill epithelial cells supply sulfur to the thiotrophic symbionts with thiotaurine and hypotaurine by TAUT. In *B. platifrons* from Sagami Bay, however, although the mussel did not contain thiotrophic symbionts, TAUT expression also increased in response to a continuous exposure to sulfide (at 1 atm)^59^, further suggesting that the involvement of TAUT in sulfide detoxification. In the current study, however, the expression level of TAUT was higher in the mantle than in the gill. Such inconsistent finding might be due to a lower sulfide level in the methane seep environment.

## Conclusion

We presented a transcriptomic database for the cold seep mussel *B. platifrons*. We highlighted a repertoire of genes involved in the innate immune system, heavy metal binding and sulfide detoxification. The expression patterns of these genes in in the gill, foot and mantle were also reported. In particular, we reported a potentially novel metal binding MT-like protein and the possibility of PC production in *B. platifrons*. Our in-depth analysis of PGRP, BD and macin demonstrated that different isoforms of the same genes may exhibit a very different tissue expression pattern. These expressional differences may be related to functional variation among isoforms. Overall, we believe that our transcriptomic data will serve as an important reference for future research on adaptation Bathymodiolid*s*and host-symbiont interaction of Bathymodiolid mussels.

## Materials and Methods

### Sample collection and preparation for Illumina sequencing

**Figure 1:**
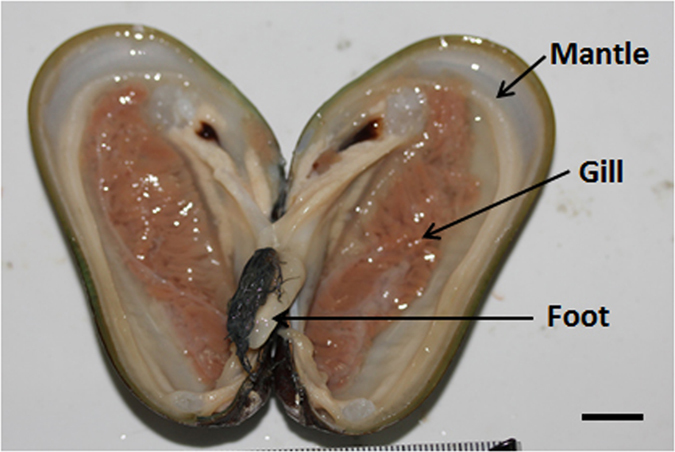
A photograph of *Bathymodiolus platifrons* with arrows showing the gill, foot and mantle. Scale bar = 1 cm.

**Figure 2:**
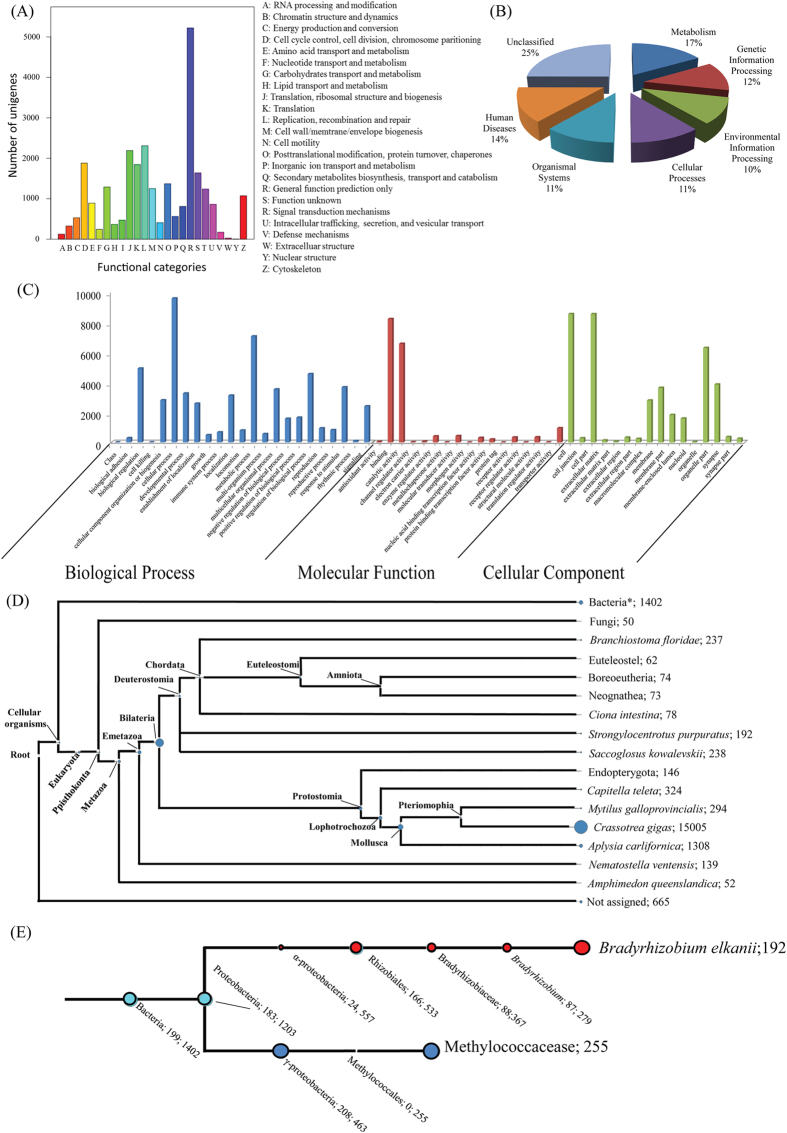
A summary of the functional annotation of *B. platifrons* transcriptome (the overall transcriptome). Functional classification of annotated transcripts by (**A**) COG, (**B**) KEGG (**C**) Gene Ontology. (**D**) LCA assignment of annotated transcripts, (**E**) LCA assignment of bacterial transcripts. The size of circle is proportional to the number of transcript assigned to the taxon. The number behind each taxon name indicates the number of transcript assigned.

**Figure 3:**
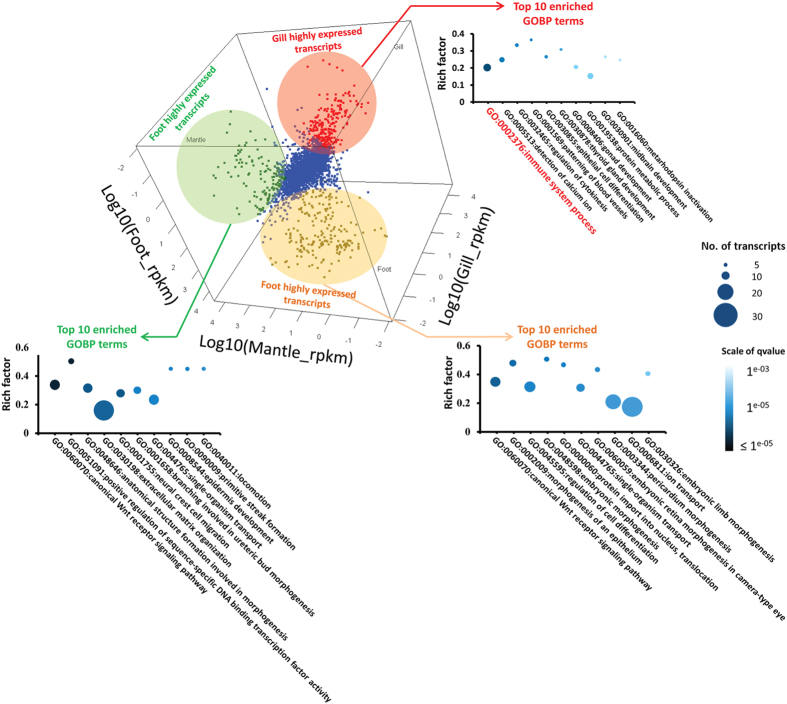
Enrichment analysis of transcripts predominantly expressed in the gill, foot and mantle. In the 3D scatterplot, transcripts predominantly expressed in the gill are red dots and highlighted in pale red circle; transcripts predominantly expressed in the foot are orange dots and highlighted in pale orange circle; transcripts predominantly expressed in the mantle were marked as green dot and highlighted in pale green circle. The top10 most enriched GO Biological Process terms for each tissue specific dominant transcript list were indicated by arrows of the corresponding color.

**Figure 4:**
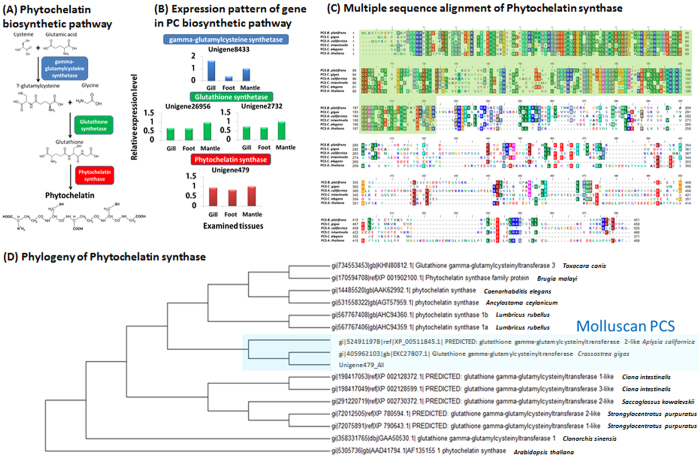
*B. platifrons* phytochelatin synthase. (**A**) The biosynthetic pathway of phytochelatin and (**B**) the expression pattern of genes involved in phytochelatin biosynthesis. (**C**) Protein sequence alignment of Unigene479_All to phytochelatin synthase/Glutathione gamma-glutamylcysteinyltransferase from the Pacific Oyster *Crassostrea gigas* (EKC27807.1), the nametode *Caenorhabditis elegans* (AAK62992.1), the sea hares *Aplysia carlifornica* (XP_005110845.1), the tunicate *Ciona intestinalis* (XP_005110845.1), and the mode plant species *Arabidopsis thaliana* (AAD41794.1). The highly conserved N-terminal phytochelatin synthase domain was highlighted in pale green box. (**D**) Maximum likelihood phylogenetic tree of phytochelatin synthase with 1000 boostrap replications. Molluscan PCS cluster were highlighted in pale blue box.

**Figure 5:**
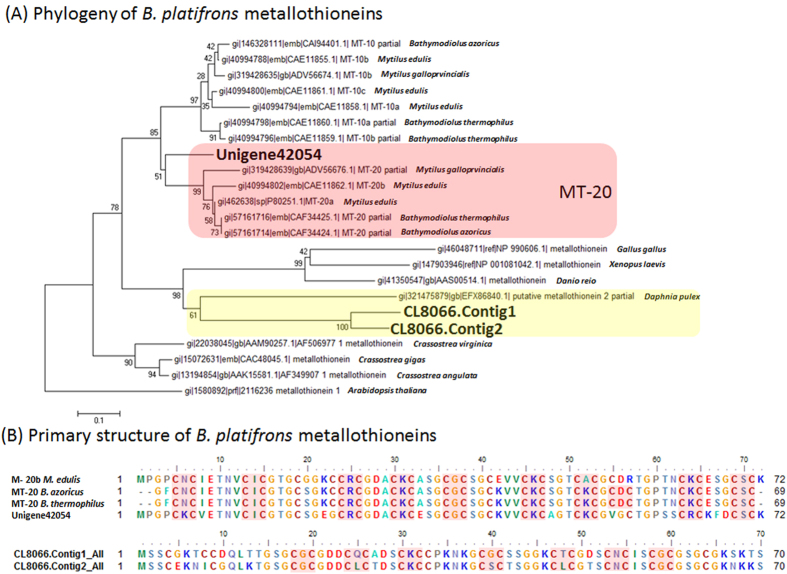
Sequence analysis of metallothioneins (MT) detected in *B. platifrons* transcriptome. (**A**) A maximum likelihood tree of MT with 1000 boostrap replications. The MT-20 clade, where Unigene42054_All situated, was highlighted in pale red box; CL2066.Contig1 and CL2066.Contig2, which clustered with *Daphnia pulex* MT, were highlighted in yellow box. (**B**) Sequence alignment of, in the upper part, Unigene42054_All with MT-20 from *Mytilus edilus* (CAE11862.1)*, Bathymodiolus azoricus* (CAF34424.1) and *B. thermophiles* (CAF34425.1), and, in the lower part, CL2066.Contig1 and CL2066.Contig2. Note that the Cys-Xaa-Cys motifs in the MT sequences were highlighted in pale red boxes.

Mussel samples were collected on June 19, 2013 using the manned submersible Jiaolong. The sampling site was a cold seep located at a depth of 1122 m on the continental slope of the South China Sea (22° 06.921′ N, 119° 17.131′ E). Upon arrival at the sea surface, the gill, the foot and the mantle tissues (Fig. 1) from three specimens of *B. platifrons* were dissected and stored individually in RNAlater (Life Technologies, USA). The shell lengths of the three specimens were 7.4 cm, 8.1 cm and 7.6 cm. One of the three *B. platifrons* was genotyped by sequencing the mitochondrial genes COI and NADH4 using previously reported primers^15^. These sequences were deposited in NCBI with the accession numbers KJ174328 and KJ174329. Around 1 g of gill, 4 g of adductor foot muscle and 4 g of mantle tissues from each mussel were used for RNA extraction. Each tissue sample was placed into a sterile (180 °C overnight) mortar that had been pre-chilled with liquid nitrogen. Additional liquid nitrogen was poured onto the tissue sample. A pestle pre-chilled in liquid nitrogen was used to crack the frozen tissues into fine powder. TRIzol reagent (Invitrogen, USA) was then added and the sample was mixed using the pestle. RNA extraction was performed according to the manufacturer’s protocol excluding the RNA precipitation step where 1/2 volume of 3 M sodium acetate and 1/2 volume of pre-chilled isopropanol were added to the retrieved aqueous phase solution. The RNA pellets were dissolved in diethylpyrocarbonate (DEPC)-treated water and stored in −80 °C until cDNA synthesis. Total RNA (10 μg) from each tissue was re-precipitated by adding 1/5 volume of 10 M lithium chloride at −20 °C for one hour. The RNA pellets were re-dissolved in DEPC-treated water. mRNA was isolated with Oligo(dT) beads and fragmented into small pieces to avoid priming bias. Using these short fragments as templates, random hexamers were used to synthesize double-stranded cDNA using SuperScript® Double-Stranded cDNA Synthesis Kit (Invitrogen, USA). The synthesized cDNA was subjected to end-repair, phosphorylation, 3′ adenylation, and ligation to sequencing adapters using Illumina Truseq RNA Sample Preparation kit v2 (Illumina, USA).

### Illumina sequencing, *de novo* assembly and functional annotation of *B. platifrons* transcriptomes

Illumina sequencing, *de novo* assemby and functional annotation were performed by the Beijing Genomics Institute, Hong Kong. Illumina sequencing was performed using a HiSeq™ 2000 (Illumina, USA) in paired-end mode with a read length of 100 bp. Five Gbp clean data was targeted for each tissue library. Briefly, cDNA libraries from the three tissues were sequenced separately. Raw reads with adaptor sequences, or with more than 5% unknown nucleotides, or with more than 20% low quality bases (base quality ≤10) were removed using the NGS QC toolkit package (version 2.3)^60^. All of the clean reads from each examined tissue were firstly assembled independently using Trinity assembly algorithm^61^. The assembling parameters were set as “seqType = fq, min_contig_length = 100, min_kmer_cov = 2”, with the rest being default parameter. All the sequences from all three tissue specific transcriptomes were then taken into further process of redundancy removing using CD-HIT-EST v4.6^62^ with a sequence identity threshold of 99% in every 1000 bp. Gene family clustering analysis was performed using MC-UPGMA v1.0.0^63^, where non-reduntant sequences with similarity between each other exceeding 70% were assigned into one gene family cluster. The prefix “CL” was given to these transcript sequences, e.g. CL1190.Contig1_All, CL1190.Contig2_All, CL1190.Contig3, etc. Non-redundant sequences that did not form any cluster remain as singletons and their prefix remained as Unigene.

### Functional annotation and protein coding sequence prediction

All the transcript sequences were searched against protein databases including NCBI non-redundant (nr) database (as of October, 2013), Swiss-Prot, KEGG and COG (e-value < 0.00001) using BLASTx (Version: BLAST-2.2.28+)^64^. Gene Ontology terms (as of October 20, 2013) information for the annotated transcripts were retrieved from the NCBI database (as of 20^th^ October, 2013)^65^. Taxonomical assignment of annotated transcripts was performed using the LCA assignment algorithm in MEGAN v5.2.3)^66^ based on the top 20 hits for each transcript in the nr database. For the protein coding sequence prediction, BLASTx alignment results were used to decide the frame direction and the coding region of the transcript. For transcripts that could not be orientated by BLASTx alignment, ESTScan was performed. The coding region of all of the transcripts was then translated into amino acid sequences based on the standard codon table. Protein domain discovery was performed using the software InterProScan 5 (v5.8–49)^67^, with Pfam as the database and an evalue cutoff ≤0.01. To evaluate the comprehensiveness of the current *B. platifrons* transcriptome assembly, we downloaded the 458 single-copy genes (core proteins) from the Core Eukaryotic Gene Mapping Approach (CEGMA) dataset^16^. We used tBLASTn with a evalue cut-off of 10^−3^ to assay the completeness of both assembly. The same procedure was conducted to assess the completeness of a decapod genome^68^.

### Expression level and differential expression analysis

Clean reads from each tissue specific read library were aligned to the whole body transcriptome using Bowtie2^69^. The number of aligned reads per transcript was counted using SAMtools (v2.0.1)^70^. Transcript expression levels in each tissue are presented as FPKM (fragments per kilobase of transcript per million mapped reads)^71^. To investigate the dominant gene function in each tissue, transcripts were sorted based on their total expression level (summation of expression value in all three examined tissues). Cumulative hypergeometric distribution tests were conducted to extract significantly over-represented KEGG terms in the list of the top 5% of the expressed transcripts^72^. Enrichment for a functional annotation (such as KEGG or GO) term was determined based on the P value ≤ 0.001. The degree of enrichment of an enriched term was indicated as a Rich factor, which was calculated as the number of transcripts with the enriched term in the list divided by the total number of transcripts with the enriched term. The closer the rich factor to 1, the higher the fold-enrichment of the enriched term.

As quantification accuracy is positively correlated with sequence depth^17^, prior to the differential expression analysis, transcripts that have less than 750 clean reads (equivalent to 5 ppm total mapped reads) mapped to the three examined tissues were filtered out. This resulted in 7,304 transcripts for differential expression analysis. Although such transcript filtering parameter was far more stringent comparing to a transcriptomic study conducted using the mussel *Perna viridis*^73^, applying such parameter can ensure high quantification accuracy among abundantly expressed transcripts. Similar filtering approach (10 ppm total mapped reads as cutoff) was adopted in a coral transcriptomic study^33^. Three pairwise comparisons were performed: Gill vs Foot, Gill vs Mantle and Foot vs Mantle. In each comparison, the log2 RPKM ratios of each transcript were calculated. The mean and standard deviation (SD) of the log2(ratio) in each comparison were then computed. To ensure a high accuracy in identification of differentially expressed transcripts, differential expressions were defined as 2 SD from the mean log2(ratio) value in each pairwise comparison. Such stringent criterion for identification of differentially expressed genes is rather common in microarray-based studies^74^,^75^. GO Biological function (GOBP) terms enrichment analysis in these tissue specific dominant gene lists were performed using cumulative hypergeometric distribution tests as described. Enrichment for a GOBP term was determined based on the P value ≤ 0.0001. The Rich factor of an enriched term was calculated as described.

### Validation of differentially expressed genes by semi-quantitative real-time PCR

**Figure 6:**
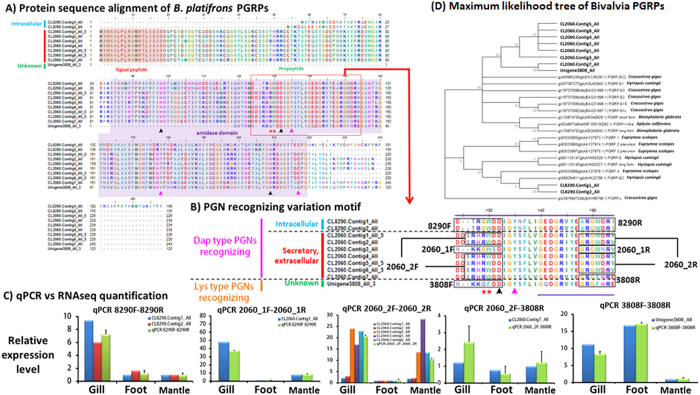
Sequence analysis of peptidoglycan recognition protein isoforms detected in *B. platifrons* transcriptome. (**A**) Sequence alignment of all PGRP transcripts in *B. platifrons* transcriptome. N-terminal signal peptides were highlighted in pale red box; propeptide sequences were highlighted in pale green box; amidase domains were highlighted in pale purple box. Black arrows indicated residues essential for zinc-binding function. Purple arrows indicated residues essential for hydrolytic amidase activity. Red asters indicated the two residues that determine the binding selectivity of PGRPs to Lys-type and Dap-type PGNs. (**B**) The PGN recognizing variation motif as signified by red outline box in (**A**). The black, purple arrows and red asters indicated the same functional residues as stated above. Note the G or RW residues are selective to Dap-type PGN while QF residues are selective to Lys-type PGN. Gene specific primer pairs targeting different PGRP isoforms was indicated in black boxes. (**C**) RNAseq quantifications versus qRT-PCR results. All qRT-PCR results were shown in light green bar in all charts while other color bars indicated the RNAseq quantification of different PGRP transcripts in different tissues. (**D**) Maximum likelihood tree of Bivalve PGRPs with 1000 boostrap replications.

To evaluate the reliability of our differential expression analysis, primer pairs specific to 30 selected transcripts, as well as to PGRP mentioned in the result part (see Fig. 6), were designed (see result part on the criteria of selection of these transcripts) for qRT-PCR assay. The 18S rRNA transcript was used as an internal standard within each sample. All gene specific primer sequences are listed in Table S7.

The cDNA samples prepared for qRT-PCR assays were derived from the three specimens that were used for preparing cDNA for Illumina sequencing. Briefly, total RNA from each specimen was prepared using TRIzol as described above. DNA contaminant was removed using the Turbo DNA-free kit (Ambion, USA). The double-stranded cDNA was synthesized by using the Superscript double-stranded cDNA synthesis kit (Invitrogen, USA) with the random hexamer primer according to the manufactory’s protocol. For each transcript, duplicated qRT-PCR assays were performed on each tissue from each mussel specimen (i.e. 3 biological specimens X 2 technical repeats per tissue). All qRTPCR assays were carried out using iTaqSYBR Green Supermix with ROX (BioRad Life Science, United States) and were run on a Stratagene mx3000p PCR machine (Agilent Technologies, Santa Clara, CA, United States). The results were normalized using the housekeeping gene 18S. The relative expression levels were calculated using the 2-ΔΔCT method^76^. To compare the qRT-PCR result against RNAseq quantification of the selected transcripts, the differential expression level between the gill and the foot (Gill-to-Foot ratio), the gill and the mantle (Gill-to-Mantle ratio), and the foot and the mantle (Foot-to-Mantle ratio) from both quantification methods were calculated and then log2 transformed. For each comparison, the log2 transformed differential expression level of each transcript from both quantification methods were plotted on a scatterplot and the Pearson’s r (R^2^) value was calculated by excel.

### Sequence analysis

All sequence alignment and phylogenetic tree construction were performed in the software MEGA 6^77^. Sequence alignment of PGRPs, PCSs and MTs was performed by the built-in alignment program MUSCLE^78^. Maximum likelihood tree of MTs, PCS and Bivalvia PGRPs were constructed based on a G + I Jones-Taylor-Thornton (JTT) model, with 1000 boostraps. Prediction of signal peptide and propeptide sequences of PGRPs was performed by ProP 1.0 server^79^.

## Additional Information

**Accession Codes**: The raw sequencing data has been submitted to the Short Read Archive (SRA) of NCBI under the accession number SRP035485.

**How to cite this article**: Wong, Y. H. *et al.* High-throughput transcriptome sequencing of the cold seep mussel *Bathymodiolus platifrons*. *Sci. Rep.* **5**, 16597; doi: 10.1038/srep16597 (2015).

## Supplementary Material

Supplementary Information

Supplementary Data File 1

Supplementary Data File 2

## Figures and Tables

**Table 1:** Summary of assembling and functional annotation of *B. platifrons* transcriptome.

*De novo* assembly by Trinity	
Total base (bp)	65,038,187
Total number of transcripts	96,683
Number of singletons	68,714
Number of gene clusters	27,969
Mean length of transcripts (bp)	673
N50 (bp)	982
Transcripts size range (bp)	200–24,272
**Functional annotation**	
Total number of transcripts annotated by public databases	40,935
NCBI non-redundant (nr) database (e-value < 1 e^−5^)	37,039
SwissProt (e-value < 1 e^−5^)	27,690
KEGG	23,524
COG	12,036
Gene Ontology	14,656
Coding sequence prediction	
CDS predicted from BLAST results	37,376
CDS predicted by ESTScan	7,257
Total CDS predicted	44,633
Mean length of CDS (aa)	213

**Table 2:** Identification of differentially expressed transcripts in each pairwise comparison.

	Pairwise comparison		
	Gill-to-Foot	Gill-to-Mantle	Foot-to-Mantle
log2(ratio) mean	0.05	0.06	−0.02
S.D.	2.55	2.22	1.72
2 S.D.	5.09	4.44	3.43
log2(ratio) cutoff for transcripts that were			
highly expressed in the gill	5.14 (mean + 2 S.D.)	4.50 (mean + 2 S.D.)	NA
highly expressed in the foot	−5.04 (mean − 2 S.D.)	NA	3.41 (mean + 2 S.D.)
highly expressed in the mantle	NA	−4.37 (mean − 2 S.D.)	−3.45 (mean − 2 S.D.)
