# Supplementary material for: High-throughput transcriptome sequencing of the cold seep mussel Bathymodiolus platifrons
Source: Sci Rep. 2015 Nov 23;5:16597. doi: 10.1038/srep16597 (PMC4655397; doi:10.1038/srep16597)
Supplement: Supplementary Information [file srep16597-s1.doc]

**Supporting information**

**High-throughput transcriptome sequencing of the cold seep mussel *Bathymodiolus platifrons***

Yue Him Wong^1*^, Jin Sun^2*^, Li Sheng He^3^, Lian Guo Chen^1^, Jian-Wen Qiu^2#^, Pei-Yuan Qian^1#^

^*^These authors contributed equally to this study

^#^Corresponding authors

^1^ Division of Life Science, School of Science, the Hong Kong University of Science and Technology, Hong Kong S.A.R
^2^Department of Biology, Hong Kong Baptist University, Hong Kong S.A.R

^3^Sanya Institute of Deep-sea Science and Engineering, Chinese Academy of Sciences, Hainan, the People Republic of China

**Table S1: Summary of Illumina sequencing**

| Samples | Total  Raw Reads | Total  Clean Reads | Total  Clean Nucleotides (nt) | Q20  percentage | N  percentage | GC  percentage |
| --- | --- | --- | --- | --- | --- | --- |
| Gill | 59,916,572 | 53,293,262 | 4,796,393,580 | 98.21% | 0.01% | 38.22% |
| Foot | 56,751,368 | 52,516,182 | 4,726,456,380 | 98.15% | 0.01% | 40.17% |
| Mental | 60,783,656 | 55,144,494 | 4,963,004,460 | 98.26% | 0.01% | 38.82% |

**Table S2: the full list of enriched KEGG pathway in the list of the top 10% transcripts**

| KEGG pathway | No. of EST with the respective GOBP term in top10% list | Pvalue | Qvalue |
| --- | --- | --- | --- |
| Oxidative phosphorylation | 68 | 0 | 0 |
| Ribosome | 80 | 0 | 0 |
| Autoimmune thyroid disease | 1 | 0 | 0 |
| Parkinson's disease | 64 | 0 | 0 |
| Pathogenic Escherichia coli infection | 45 | 5.7E-14 | 4.64E-11 |
| Huntington's disease | 87 | 4.64E-11 | 4.8E-11 |
| Spliceosome | 71 | 4.8E-11 | 7.91E-11 |
| Phagosome | 68 | 7.91E-11 | 2.01E-10 |
| Alzheimer's disease | 88 | 2.01E-10 | 6.54E-09 |
| RNA transport | 47 | 6.54E-09 | 2.51E-06 |
| Cardiac muscle contraction | 14 | 2.51E-06 | 0.000101 |
| Collecting duct acid secretion | 9 | 0.000101 | 0.000114 |
| mRNA surveillance pathway | 21 | 0.000114 | 0.000147 |
| Biosynthesis of unsaturated fatty acids | 12 | 0.000147 | 0.000147 |
| Systemic lupus erythematosus | 12 | 0.000147 | 0.000185 |
| Antigen processing and presentation | 10 | 0.000185 | 0.000202 |
| Shigellosis | 29 | 0.000202 | 0.000256 |
| Vibrio cholerae infection | 23 | 0.000256 | 0.000291 |
| Epstein-Barr virus infection | 53 | 0.000291 | 0.000428 |
| Rheumatoid arthritis | 13 | 0.000428 | 0.000847 |

**Table S3: the full list of enriched GOBP terms in the list of top10% transcripts**

| **GO number** | **GO description** | **No. of EST with the respective GOBP term in top10% list** | **P-value** | **Q-value** |
| --- | --- | --- | --- | --- |
| GO:0006413 | translational initiation | 41 | 0 | 0 |
| GO:0006414 | translational elongation | 33 | 0 | 0 |
| GO:0006614 | SRP-dependent cotranslational protein targeting to membrane | 27 | 0 | 0 |
| GO:0051258 | protein polymerization | 25 | 0 | 0 |
| GO:0019083 | viral transcription | 23 | 0 | 0 |
| GO:0006415 | translational termination | 23 | 0 | 0 |
| GO:0000184 | nuclear-transcribed mRNA catabolic process, nonsense-mediated decay | 28 | 2.89E-15 | 6.11E-15 |
| GO:0051084 | 'de novo' posttranslational protein folding | 17 | 6.11E-15 | 2.92E-14 |
| GO:0002479 | antigen processing and presentation of exogenous peptide antigen via MHC class I, TAP-dependent | 13 | 2.92E-14 | 4.32E-14 |
| GO:0006184 | GTP catabolic process | 44 | 4.32E-14 | 3.84E-11 |
| GO:0007017 | microtubule-based process | 18 | 3.84E-11 | 5.84E-11 |
| GO:0006521 | regulation of cellular amino acid metabolic process | 11 | 5.84E-11 | 6.69E-11 |
| GO:0009792 | embryo development ending in birth or egg hatching | 22 | 6.69E-11 | 1.75E-10 |
| GO:0051436 | negative regulation of ubiquitin-protein ligase activity involved in mitotic cell cycle | 13 | 1.75E-10 | 3.45E-09 |
| GO:0051533 | positive regulation of NFAT protein import into nucleus | 10 | 3.45E-09 | 3.52E-08 |
| GO:0006977 | DNA damage response, signal transduction by p53 class mediator resulting in cell cycle arrest | 12 | 3.52E-08 | 3.87E-08 |
| GO:0016061 | regulation of light-activated channel activity | 9 | 3.87E-08 | 5.31E-08 |
| GO:0016060 | metarhodopsin inactivation | 9 | 5.31E-08 | 1.31E-07 |
| GO:0016062 | adaptation of rhodopsin mediated signaling | 9 | 1.31E-07 | 1.67E-07 |
| GO:0060278 | regulation of ovulation | 9 | 1.67E-07 | 1.84E-07 |
| GO:0046676 | negative regulation of insulin secretion | 9 | 1.84E-07 | 1.84E-07 |
| GO:0000086 | G2/M transition of mitotic cell cycle | 18 | 1.84E-07 | 1.84E-07 |
| GO:0051437 | positive regulation of ubiquitin-protein ligase activity involved in mitotic cell cycle | 13 | 1.84E-07 | 1.84E-07 |
| GO:0031101 | fin regeneration | 7 | 1.84E-07 | 1.84E-07 |
| GO:0016071 | mRNA metabolic process | 12 | 1.84E-07 | 2.39E-07 |
| GO:0007099 | centriole replication | 9 | 2.39E-07 | 3.96E-07 |
| GO:0006457 | protein folding | 20 | 3.96E-07 | 6E-07 |
| GO:0007052 | mitotic spindle organization | 14 | 6E-07 | 7.7E-07 |
| GO:0008049 | male courtship behavior | 10 | 7.7E-07 | 1.64E-06 |
| GO:0015991 | ATP hydrolysis coupled proton transport | 9 | 1.64E-06 | 2.08E-06 |
| GO:0051383 | kinetochore organization | 9 | 2.08E-06 | 2.12E-06 |
| GO:0031145 | anaphase-promoting complex-dependent proteasomal ubiquitin-dependent protein catabolic process | 13 | 2.12E-06 | 2.83E-06 |
| GO:0022900 | electron transport chain | 7 | 2.83E-06 | 3.01E-06 |
| GO:2001020 | regulation of response to DNA damage stimulus | 9 | 3.01E-06 | 3.01E-06 |
| GO:0061418 | regulation of transcription from RNA polymerase II promoter in response to hypoxia | 6 | 3.01E-06 | 3.02E-06 |
| GO:0005513 | detection of calcium ion | 10 | 3.02E-06 | 8.5E-06 |
| GO:0006898 | receptor-mediated endocytosis | 14 | 8.5E-06 | 8.9E-06 |
| GO:0051489 | regulation of filopodium assembly | 9 | 8.9E-06 | 1.22E-05 |
| GO:0000398 | mRNA splicing, via spliceosome | 23 | 1.22E-05 | 1.73E-05 |
| GO:0002119 | nematode larval development | 13 | 1.73E-05 | 1.84E-05 |
| GO:0040007 | growth | 11 | 1.84E-05 | 2.28E-05 |
| GO:0042981 | regulation of apoptotic process | 23 | 2.28E-05 | 2.63E-05 |
| GO:0046331 | lateral inhibition | 15 | 2.63E-05 | 3.47E-05 |
| GO:0040039 | inductive cell migration | 6 | 3.47E-05 | 4.89E-05 |
| GO:0051301 | cell division | 19 | 4.89E-05 | 4.92E-05 |
| GO:0030048 | actin filament-based movement | 10 | 4.92E-05 | 4.96E-05 |
| GO:0006096 | glycolysis | 8 | 4.96E-05 | 5.18E-05 |
| GO:0045454 | cell redox homeostasis | 7 | 5.4E-05 | 9.96E-05 |
| GO:0000209 | protein polyubiquitination | 13 | 9.96E-05 | 0.000101 |
| GO:0060766 | negative regulation of androgen receptor signaling pathway | 5 | 0.000101 | 0.000106 |
| GO:0000084 | S phase of mitotic cell cycle | 13 | 0.000106 | 0.000112 |
| GO:0072499 | photoreceptor cell axon guidance | 9 | 0.000112 | 0.000122 |
| GO:0040035 | hermaphrodite genitalia development | 7 | 0.000122 | 0.000141 |
| GO:0006508 | proteolysis | 27 | 0.000141 | 0.000164 |
| GO:0019048 | virus-host interaction | 11 | 0.000164 | 0.000175 |
| GO:0016032 | viral reproduction | 11 | 0.000175 | 0.000205 |
| GO:0016044 | cellular membrane organization | 8 | 0.000205 | 0.00021 |
| GO:0000022 | mitotic spindle elongation | 5 | 0.00021 | 0.000217 |
| GO:0000281 | cytokinesis after mitosis | 5 | 0.000217 | 0.000238 |
| GO:0006367 | transcription initiation from RNA polymerase II promoter | 14 | 0.000238 | 0.00026 |
| GO:0006369 | termination of RNA polymerase II transcription | 8 | 0.00026 | 0.00026 |
| GO:0045727 | positive regulation of translation | 6 | 0.00026 | 0.000277 |
| GO:0042254 | ribosome biogenesis | 7 | 0.000277 | 0.000316 |
| GO:0051603 | proteolysis involved in cellular protein catabolic process | 7 | 0.000316 | 0.000324 |
| GO:0007616 | long-term memory | 9 | 0.000324 | 0.000324 |
| GO:0071688 | striated muscle myosin thick filament assembly | 5 | 0.000324 | 0.000422 |
| GO:0000413 | protein peptidyl-prolyl isomerization | 8 | 0.000422 | 0.000436 |
| GO:0019221 | cytokine-mediated signaling pathway | 7 | 0.000436 | 0.000441 |
| GO:0006364 | rRNA processing | 10 | 0.000441 | 0.000466 |
| GO:0007219 | Notch signaling pathway | 9 | 0.000466 | 0.000466 |
| GO:0007140 | male meiosis | 4 | 0.000466 | 0.000502 |
| GO:0007264 | small GTPase mediated signal transduction | 24 | 0.000502 | 0.000555 |
| GO:0043388 | positive regulation of DNA binding | 5 | 0.000555 | 0.000576 |
| GO:0050434 | positive regulation of viral transcription | 5 | 0.000576 | 0.00059 |
| GO:0008543 | fibroblast growth factor receptor signaling pathway | 9 | 0.00059 | 0.000658 |
| GO:0048011 | neurotrophin TRK receptor signaling pathway | 12 | 0.000658 | 0.00084 |
| GO:0030182 | neuron differentiation | 8 | 0.00084 | 0.000889 |
| GO:0007067 | mitosis | 14 | 0.000889 | 0.000889 |
| GO:0015031 | protein transport | 25 | 0.000889 | 0.000889 |
| GO:0006730 | one-carbon metabolic process | 4 | 0.000889 | 0.000889 |

Table S4: RNAseq quantification and qRT-PCR results of selected tissue dominant transcripts

|  | **Transcript no.** | **Gene length (bp)** | **total mapped reads** | **Nr annotation** | **RNAseq quantification** | | | **qRT-PCR** | | | | | |
| --- | --- | --- | --- | --- | --- | --- | --- | --- | --- | --- | --- | --- | --- |
|  |  |  |  |  | **Log2(G/F)** | **Log2(G/M)** | **Log2(F/M)** | **Log2(G/F)** | **S.D.** | **Log2(G/M)** | **S.D.** | **Log2(F/M)** | **S.D.** |
| **Gill dominant** | Unigene51974_All | 1,182 | 2,812 | gi|405959429|gb|EKC25470.1|hypothetical protein CGI_10008900 [*Crassostrea gigas*] | **11.44** | **9.96** | **-1.48** | **9.29** | 0.52 | **8.25** | 0.43 | **0.21** | 0.37 |
|  | Unigene36448_All | 1,254 | 101,130 | gi\|405972828\|gb\|EKC37576.1\|Collagen alpha-5(VI) chain [*Crassostrea gigas*] | **9.69** | **7.84** | **-1.85** | **6.64** | 0.21 | **6.38** | 0.12 | **-1.62** |  |
|  | CL5231.Contig2_All | 1,673 | 17,808 | gi\|405958435\|gb\|EKC24563.1\|Globin [*Crassostrea gigas*] | **9.19** | **7.59** | **-1.60** | **5.76** | 0.10 | **6.34** | 0.39 | **-0.96** | 0.45 |
|  | CL79.Contig4_All | 1,904 | 1,869 | gi\|405957318\|gb\|EKC23539.1\|Heat shock 70 kDa protein 12B [*Crassostrea gigas*] | **4.31** | **5.57** | **1.27** | **5.45** | 0.31 | **5.32** | 0.45 | **0.96** | 0.34 |
|  | CL3371.Contig1_All | 529 | 4,197 | ---- | **5.94** | **5.41** | **-0.53** | **4.79** | 0.19 | **4.06** | 0.43 | **0.27** | 0.39 |
|  | CL5089.Contig1_All | 538 | 1,587 | gi\|405946277\|gb\|EKC17600.1\|hypothetical protein CGI_10000492 [*Crassostrea gigas*] | **6.09** | **5.14** | **-0.96** | **4.30** | 0.09 | **5.62** | 0.45 | **0.09** | 0.23 |
|  | CL3148.Contig1_All | 2,638 | 4,126 | gi\|405971561\|gb\|EKC36393.1\|Zonadhesin [*Crassostrea gigas*] | **7.45** | **4.25** | **-3.20** | **4.18** | 0.10 | **4.00** | 0.32 | **-3.83** | 0.27 |
|  | Unigene43926_All | 363 | 1,142 | gi\|291227041\|ref\|XP_002733496.1\|PREDICTED: matriptase-like, partial [Saccoglossus kowalevskii] | **6.45** | **4.03** | **-2.41** | **3.99** | 0.18 | **4.02** | 0.27 | **-1.44** | 0.21 |
|  | CL5388.Contig1_All | 1,703 | 969 | gi\|405954756\|gb\|EKC22108.1\|hypothetical protein CGI_10002767 [*Crassostrea gigas*] | **5.04** | **3.56** | **-1.48** | **3.57** | 0.19 | **4.34** | 0.13 | **-1.84** | 0.60 |
|  | Unigene5652_All | 628 | 1,508 | ---- | **5.89** | **3.10** | **-2.79** | **3.50** | 0.27 | **3.60** | 0.09 | **-2.30** | 0.06 |
| **Foot dominant** | CL10676.Contig1_All | 732 | 6,535 | gi|295293387|gb|ADF87943.1|C-type lectin 5 [*Azumapecten farreri*] | **-3.82** | **2.07** | **5.89** | **-3.57** | 0.31 | **1.13** | 0.09 | **7.29** | 0.12 |
|  | Unigene9278_All | 2,385 | 4,983 | gi\|405951425\|gb\|EKC19339.1\|hypothetical protein CGI_10008841 [*Crassostrea gigas*] | **-5.35** | **0.37** | **5.73** | **-4.67** | 0.45 | **0.60** | 0.02 | **6.64** | 0.22 |
|  | CL9068.Contig1_All | 6,622 | 290,979 | gi\|198419275\|ref\|XP_002122650.1\|PREDICTED: SCO-spondin-like [*Ciona intestinalis*] | **-6.20** | **-0.15** | **6.05** | **-4.98** | 0.34 | **0.34** | 0.08 | **5.76** | 0.06 |
|  | Unigene36314_All | 1,202 | 874 | gi\|405976227\|gb\|EKC40740.1\|Annexin A6 [*Crassostrea gigas*] | **-4.34** | **-0.40** | **3.93** | **-3.21** | 0.09 | **0.06** | 0.09 | **5.45** | 0.12 |
|  | Unigene5460_All | 1,131 | 1,960 | ---- | **-4.36** | **-1.31** | **3.05** | **-4.98** | 0.12 | **-1.30** |  | **4.79** | 0.07 |
|  | CL163.Contig2_All | 231 | 1,106 | gi\|198419275\|ref\|XP_002122650.1\|PREDICTED: SCO-spondin-like [*Ciona intestinalis*] | **-10.12** | **-1.91** | **8.21** | **-10.35** | 0.37 | **0.04** | 0.36 | **4.30** | 0.07 |
|  | Unigene12641_All | 953 | 33,400 | gi\|326435260\|gb\|EGD80830.1\|hypothetical protein PTSG_11726 [*Salpingoeca* sp. ATCC 50818] | **-11.45** | **-2.83** | **8.62** | **-9.23** | 0.20 | **-2.30** | 0.22 | **4.18** | 0.07 |
|  | CL2829.Contig2_All | 853 | 7,868 | ---- | **-8.08** | **-4.48** | **3.60** | **-4.67** | 0.13 | **-5.45** | 0.07 | **3.99** | 0.31 |
|  | CL3424.Contig1_All | 630 | 59,311 | ---- | **-7.84** | **-5.91** | **1.93** | **-4.73** | 0.36 | **-6.36** | 0.34 | **3.57** | 0.19 |
|  | CL40.Contig5_All | 2,597 | 8,700 | gi\|1408192\|gb\|AAB03660.1\|myosin heavy chain [*Placopecten magellanicus*] | **-9.74** | **-6.85** | **2.88** | **-8.54** | 0.31 | **-7.32** | 0.16 | **3.50** | 0.09 |
| **Mantle dominant** | Unigene24812_All | 752 | 25,981 | gi|325504479|emb|CBX41739.1|putative C1q domain containing protein MgC1q90 [*Mytilus galloprovincialis*] | **-0.25** | **-3.85** | **-3.60** | **0.23** | 0.12 | **-3.72** | 0.10 | **-3.13** | 0.16 |
|  | CL10404.Contig1_All | 1,327 | 22,490 | gi\|405953320\|gb\|EKC21006.1\|hypothetical protein CGI_10004855 [*Crassostrea gigas*] | **-1.01** | **-4.36** | **-3.35** | **-1.41** | 0.22 | **-4.65** | 0.09 | **-3.66** | 0.02 |
|  | CL541.Contig3_All | 1,935 | 1152 | gi\|2196556\|dbj\|BAA20456.1\|troponin T [*Mizuhopecten yessoensis*] | **-2.13** | **-4.85** | **-2.72** | **-2.84** | 0.06 | **-4.25** | 0.34 | **-3.83** | 0.12 |
|  | CL40.Contig4_All | 6,368 | 87,477 | gi\|6682319\|emb\|CAB64662.1\|myosin heavy chain [*Mytilus galloprovincialis*] | **-3.25** | **-5.47** | **-2.22** | **-3.84** | 0.12 | **-4.07** | 0.10 | **-4.00** | 0.23 |
|  | CL3.Contig28_All | 3,311 | 2,467 | gi\|405966303\|gb\|EKC31603.1\|hypothetical protein CGI_10009847 [*Crassostrea gigas*] | **-3.85** | **-5.97** | **-2.12** | **-2.89** | 0.16 | **-6.46** | 0.18 | **-4.81** | 0.09 |
|  | Unigene39009_All | 527 | 1,192 | gi\|405957680\|gb\|EKC23874.1\|Inter-alpha-trypsin inhibitor heavy chain H3 [*Crassostrea gigas*] | **-4.59** | **-7.09** | **-2.50** | **-4.62** | 0.02 | **-6.21** | 0.19 | **-5.08** | 0.12 |
|  | Unigene63259_All | 675 | 1,711 | ---- | **NA** | **-7.64** | **NA** | **NA** |  | **-7.35** | 0.40 | **NA** |  |
|  | CL7273.Contig1_All | 1,651 | 160,425 | ---- | **0.89** | **-8.82** | **-9.71** | **1.43** | 0.23 | **-9.99** | 0.46 | **-6.63** | 0.34 |
|  | Unigene13644_All | 1,177 | 206,283 | ---- | **-0.04** | **-9.34** | **-9.30** | **1.13** | 0.12 | **-11.66** | 0.52 | **-8.90** | 0.31 |
|  | CL11405.Contig1_All | 3,098 | 781 | gi\|405969197\|gb\|EKC34183.1\|hypothetical protein CGI_10008927 [*Crassostrea gigas*] | **NA** | **NA** | **-2.64** | **NA** |  | **NA** |  | **-4.39** | 0.06 |

Table S5: Heavy metal detoxification genes and sulfide metabolism genes

| **GeneID** | **Transcript length (bp)** | **Expression level (RPKM)** | | | **Nr annotation** | **Nr blastx score** | **Pfam domain^#^** |  |
| --- | --- | --- | --- | --- | --- | --- | --- | --- |
|  |  | **Gill** | **Foot** | **Mantle** |  |  |  |  |
| **Heavy metal ion binding** | |  |  |  |  |  |  |  |
| **Phytochelating synthase** | | | | | |  |  |  |
| Unigene479_All | 1,584 | 6.68 | 5.88 | 7.09 | gb|EKC27807.1|Glutathione gamma-glutamylcysteinyltransferase 3 [*Crassostrea gigas*] | 411 | PF05023:Phytochelatin synthase |  |
| **Glutathione synthetase** | | | | | |  |  |  |
| Unigene26956_All | 1,203 | 3.07 | 3.01 | 4.47 | gb|EKC19457.1|Glutathione synthetase [*Crassostrea gigas*] | 390.6 | PF03917:Eukaryotic glutathione synthase, ATP binding domain |  |
| Unigene2732_All | 435 | 4.82 | 4.49 | 6.54 | gb|EKC19457.1|Glutathione synthetase [*Crassostrea gigas*] | 115.2 | PF03917:Eukaryotic glutathione synthase, ATP binding domain |  |
| **Gamma-glutamylcysteine synthetase** | | | | | |  |  |  |
| Unigene8433_All | 279 | 2.77 | 0.60 | 1.67 | gb|AAV48595.2|gamma-glutamylcysteine synthetase [*Laeonereis acuta*] | 56.2 | NA |  |
| **Metallothionein** | | | | | |  |  |  |
| Unigene30334_All | 263 | 1.47 | 0.85 | 0.59 | ref|NP_990606.1|metallothionein [*Gallus gallus*] | 157.9 | PF00131:Metallothionein |  |
| Unigene42054_All | 507 | 421.14 | 190.93 | 378.73 | sp|P80251.1|MT21_MYTED | 151 | PF00131:Metallothionein |  |
| CL8066.Contig1_All | 730 | 171.63 | 187.33 | 326.11 | gb|EFX86840.1||putative metallothionein 2 [*Daphnia pulex*] | 33.5* | NA |  |
| CL8066.Contig2_All | 479 | 130.55 | 132.32 | 132.22 | No match | NA | NA |  |
| **Sulfide metabolism** | | | | | |  |  |  |
| **Sulfite oxidase** | | | | | |  |  |  |
| Unigene26503_All | 4,510 | 52.93 | 4.52 | 4.72 | gb|EKC42604.1|Putative sulfite oxidase, mitochondrial [*Crassostrea gigas*] | 699.5 | PF00173:Cytochrome b5-like Heme/Steroid binding domain | |
| CL6176.Contig1_All | 3,744 | 46.50 | 2.82 | 2.68 | gb|EKC42604.1|Putative sulfite oxidase, mitochondrial [*Crassostrea gigas*] | 541.2 | PF03404:Mo-co oxidoreductase dimerisation domain | |
| **Sulfite reductase** | | | | | |  |  |  |
| Unigene19303_All | 465 | 1.42 | 1.80 | 1.22 | ref|XP_002741788.1|PREDICTED: cytochrome P450 reductase-like [*Saccoglossus kowalevskii*] |  | PF00667:FAD binding domain |  |
| Unigene8776_All | 437 | 1.77 | 1.02 | 1.18 | gb|EKC29137.1|NADPH oxidoreductase A [*Crassostrea gigas*] | 94.7 | NA |  |
| Unigene58781_All | 235 | 1.88 | 1.90 | 0.44 | ref|XP_002741788.1|PREDICTED: cytochrome P450 reductase-like [*Saccoglossus kowalevskii*] | 87.4 | PF00175:Oxidoreductase NAD-binding domain |  |
| Unigene46612_All | 342 | 1.13 | 0.00 | 0.00 | ref|WP_027148836.1|sulfite reductase [*Methylobacter tundripaludum*] | 90.9 | PF03460:Nitrite/Sulfite reductase ferredoxin-like half domain |  |
| **Sulfide:quinone oxidoreductase, mitochondrial** | | | | | |  |  |  |
| Unigene1045_All | 1,759 | 25.93 | 4.28 | 11.97 | gb|EKC32430.1|Sulfide:quinone oxidoreductase, mitochondrial [*Crassostrea gigas*] | 540 | PF07992:Pyridine nucleotide-disulphide oxidoreductase |  |
| CL713.Contig4_All | 579 | 2.10 | 7.32 | 3.93 | gb|AEB61068.1|sulfide:quinone oxidoreductase mitochondrial-like protein, partial [*Equus caballus*] | 231.5 | NA |  |
| CL713.Contig3_All | 1,064 | 8.24 | 2.36 | 3.65 | gb|ACO13065.1|Sulfide:quinone oxidoreductase, mitochondrial precursor [*Lepeophtheirus salmonis*] | 310.8 | NA |  |
| **Taurine transporter (TAUT)** | | | | | |  |  |  |
| Unigene37617_All | 789 | 10.34 | 13.29 | 25.05 | dbj|BAI66658.1|taurine transporter [*Bathymodiolus platifrons*] | 393.7 | PF00209:Sodium:neurotransmitter symporter family |  |
| Unigene35307_All | 2,422 | 8.74 | 9.26 | 16.32 | dbj|BAI66658.2|taurine transporter [*Bathymodiolus platifrons*] | 295.8 | PF00209:Sodium:neurotransmitter symporter family |  |
| CL1972.Contig3_All | 2,457 | 3.97 | 5.63 | 7.08 | dbj|BAI66658.3|taurine transporter [*Bathymodiolus platifrons*] | 903.7 | PF00209:Sodium:neurotransmitter symporter family |  |
| CL1972.Contig2_All | 3,083 | 1.75 | 1.70 | 3.29 | dbj|BAI66658.4|taurine transporter [*Bathymodiolus platifrons*] | 408.7 | PF00209:Sodium:neurotransmitter symporter family |  |
| Unigene12952_All | 571 | 1.06 | 1.37 | 1.81 | dbj|BAD91313.1|taurine transporter [*Mytilus galloprovincialis*] | 76.6 | PF00209:Sodium:neurotransmitter symporter family |  |
| CL1972.Contig1_All | 1,457 | 0.42 | 0.57 | 1.31 | dbj|BAI66658.1|taurine transporter [*Bathymodiolus platifrons*] | 292.7 | PF00209:Sodium:neurotransmitter symporter family |  |
| CL1972.Contig6_All | 1,010 | 0.49 | 0.99 | 1.28 | dbj|BAI66658.2|taurine transporter [*Bathymodiolus platifrons*] | 475.3 | PF00209:Sodium:neurotransmitter symporter family |  |
| CL1972.Contig5_All | 1,203 | 1.01 | 1.21 | 0.99 | dbj|BAI66658.3|taurine transporter [*Bathymodiolus platifrons*] | 644 | PF00209:Sodium:neurotransmitter symporter family |  |
| Unigene3782_All | 1,070 | 0.31 | 0.89 | 0.53 | dbj|BAI66658.4|taurine transporter [*Bathymodiolus platifrons*] | 82 | NA |  |
| CL1972.Contig4_All | 1,368 | 0.24 | 0.29 | 0.34 | dbj|BAI66658.5|taurine transporter [*Bathymodiolus platifrons*] | 569.3 | PF00209:Sodium:neurotransmitter symporter family |  |
| CL671.Contig1_All | 1,070 | 0.21 | 0.21 | 0.24 | dbj|BAI66658.6|taurine transporter [*Bathymodiolus platifrons*] | 100.5 | PF00209:Sodium:neurotransmitter symporter family |  |
| CL671.Contig3_All | 905 | 0.00 | 0.12 | 0.11 | dbj|BAI66658.7|taurine transporter [*Bathymodiolus platifrons*] | 175.3 | PF00209:Sodium:neurotransmitter symporter family |  |

#Pfam domain was identified by HMM search against the pfam database, with a evalue threshold of 1e-04.

*marginal scores

**Table S6: Immune system genes**

| **Transcript no.** | **Transcript length (bp)** | **Relative expression level (rpkm)** | | | **Nr annotation** | **Pfam domain#** | **Reference** |
| --- | --- | --- | --- | --- | --- | --- | --- |
|  |  | **gill** | **foot** | **mantle** |  |  |  |
| **Peptidoglycan-recognition protein** | | | |  |  |  | Itoh & Takahashi 2009, *Crassostrea gigas* |
| CL2060.Contig1_All | 1,102 | 3.6 | 1.67 | 3.15 | gi|405960259|gb|EKC26200.1|Peptidoglycan-recognition protein SC2 [*Crassostrea gigas*] | PF01510:N-acetylmuramoyl-L-alanine amidase |  |
| CL2060.Contig2_All | 1,238 | 4.41 | 1.49 | 3.13 | gi|405960259|gb|EKC26200.1|Peptidoglycan-recognition protein SC2 [*Crassostrea gigas*] | PF01510:N-acetylmuramoyl-L-alanine amidase |  |
| CL2060.Contig3_All | 843 | 73.2 | 1.52 | 12.03 | gi|405960259|gb|EKC26200.1|Peptidoglycan-recognition protein SC2 [*Crassostrea gigas*] | PF01510:N-acetylmuramoyl-L-alanine amidase |  |
| CL2060.Contig4_All | 849 | 4.74 | 0.2 | 2.68 | gi|405960259|gb|EKC26200.1|Peptidoglycan-recognition protein SC2 [*Crassostrea gigas*] | PF01510:N-acetylmuramoyl-L-alanine amidase |  |
| CL2060.Contig5_All | 1,031 | 10.11 | 0.6 | 16.81 | gi|405960259|gb|EKC26200.1|Peptidoglycan-recognition protein SC2 [*Crassostrea gigas*] | PF01510:N-acetylmuramoyl-L-alanine amidase |  |
| CL2060.Contig6_All | 928 | 8.26 | 0.36 | 4.85 | gi|405960259|gb|EKC26200.1|Peptidoglycan-recognition protein SC2 [*Crassostrea gigas*] | PF01510:N-acetylmuramoyl-L-alanine amidase |  |
| CL2060.Contig7_All | 853 | 17.2 | 10.86 | 14.07 | gi|405960259|gb|EKC26200.1|Peptidoglycan-recognition protein SC2 [*Crassostrea gigas*] | PF01510:N-acetylmuramoyl-L-alanine amidase |  |
| CL8290.Contig1_All | 880 | 47 | 5.07 | 5 | gi|63033995|gb|AAY27974.1|peptidoglycan recognition protein 2 precursor [*Euprymna scolopes*] | PF01510:N-acetylmuramoyl-L-alanine amidase |  |
| CL8290.Contig2_All | 508 | 8.58 | 2.31 | 1.43 | gi|63033995|gb|AAY27974.1|peptidoglycan recognition protein 2 precursor [*Euprymna scolopes*] | PF01510:N-acetylmuramoyl-L-alanine amidase |  |
| Unigene3808_All | 405 | 2.86 | 4.27 | 0.26 | gi|405960259|gb|EKC26200.1|Peptidoglycan-recognition protein SC2 [*Crassostrea gigas*] | PF01511:N-acetylmuramoyl-L-alanine amidase |  |
| **Lipopolysaccharide binding protein/Bactericidal permeability increasing protein** | | | | | |  | Zhang et al. 2011, *Crassostrea gigas* |
| CL748.Contig1_All | 2,392 | 39.03 | 35.03 | 48.09 | gi|229472842|gb|ACQ72925.1|bactericidal permeability increasing protein [*Crassostrea gigas*] | PF02886:LBP / BPI / CETP family, N-termi--l domain |  |
| CL748.Contig3_All | 2,211 | 34.64 | 5.58 | 4.07 | gi|229472842|gb|ACQ72925.1|bactericidal permeability increasing protein [*Crassostrea gigas*] | PF02886:LBP / BPI / CETP family, C-termi--l domain |  |
| **Toll-like receptor** |  |  |  |  |  |  | Toubiana et al. 2013, *Mytilus  galloprovincialis;* Zhang & Zhang 2011, *Crassostrea gigas* |
| Unigene3025_All | 1,438 | 16.07 | 19.75 | 21.84 | gi|452755167|gb|AGG10805.1|toll-like receptor g precursor [*Mytilus galloprovincialis*] | PF13855:Leucine rich repeat+TIR domain |  |
| Unigene22543_All | 2,494 | 17.09 | 33.4 | 35.77 | gi|407907625|gb|AFU48615.1|toll-like receptor c [*Mytilus galloprovincialis*] | PF13516:Leucine rich repeat+TIR domain |  |
| CL8426.Contig1_All | 1,463 | 11.31 | 5.45 | 5.27 | gi|405969804|gb|EKC34755.1|Toll-like receptor 2 type-1 [*Crassostrea gigas*] | PF00560:Leucine rich repeat+TIR domain |  |
| CL5376.Contig1_All | 2,731 | 10.92 | 23.8 | 35.86 | gi|471180497|gb|AGI05199.1|toll-like receptor W precursor [*Mytilus galloprovincialis*] | PF13855:Leucine rich repeat+TIR domain |  |
| Unigene4761_All | 2,209 | 2.1 | 8.89 | 5.6 | gi|452755165|gb|AGG10804.1|toll-like receptor f precursor [*Mytilus galloprovincialis*] | PF13855:Leucine rich repeat+TIR domain |  |
| CL1298.Contig1_All | 1,303 | 13.2 | 1.46 | 1.27 | gi|471180485|gb|AGI05193.1|toll-like receptor Q precursor [*Mytilus galloprovincialis*] | PF13855:Leucine rich repeat+TIR domain |  |
| Unigene9872_All | 1,142 | 13.91 | 0.73 | 1.54 | gi|405969802|gb|EKC34753.1|Toll-like receptor 6 [*Crassostrea gigas*] | PF12799:Leucine rich repeat+TIR domain |  |
| Unigene16197_All | 1,781 | 0.22 | 5.17 | 5.29 | gi|407907625|gb|AFU48615.1|toll-like receptor c [*Mytilus galloprovincialis*] | PF13855:Leucine rich repeat+TIR domain |  |
| **Interleukin 17** |  |  |  |  |  |  | Roberts et al. 2008, *Crassostrea gigas* |
| Unigene10382_All | 877 | 0.82 | 31.94 | 37.52 | gi|440918775|gb|AGC24392.1|interleukin 17 [*Pinctada fucata x Pinctada maculata*] | PF06083:Interleukin-17 |  |
| **Tumor necrosis factor** | |  |  |  |  |  | Hughes et al. 1990, *Mytilus edilus* |
| CL5522.Contig1_All | 1,245 | 34.86 | 17.79 | 51.2 | gi|405964023|gb|EKC29547.1|Tumor necrosis factor ligand superfamily member 14 [*Crassostrea gigas*] | PF00229:TNF(Tumour Necrosis Factor) family |  |
| CL5522.Contig2_All | 1,229 | 24.72 | 19.93 | 7.7 | gi|405964023|gb|EKC29547.1|Tumor necrosis factor ligand superfamily member 14 [*Crassostrea gigas*] | PF00229:TNF(Tumour Necrosis Factor) family |  |
| Unigene20833_All | 1,135 | 22.93 | 32.39 | 26.99 | gi|405964023|gb|EKC29547.1|Tumor necrosis factor ligand superfamily member 14 [*Crassostrea gigas*] | PF00229:TNF(Tumour Necrosis Factor) family |  |
| Unigene9263_All | 3,757 | 5.9 | 8.73 | 8.15 | gi|405974612|gb|EKC39243.1|Tumor necrosis factor ligand superfamily member 11 [*Crassostrea gigas*] | PF00229:TNF(Tumour Necrosis Factor) family |  |
| CL9145.Contig1_All | 1,190 | 11.26 | 3.19 | 4.7 | gi|405970232|gb|EKC35160.1|Tumor necrosis factor ligand superfamily member 10 [*Crassostrea gigas*] | PF00229:TNF(Tumour Necrosis Factor) family |  |
| Unigene5693_All | 557 | 3.37 | 6.61 | 2.88 | gi|405970232|gb|EKC35160.1|Tumor necrosis factor ligand superfamily member 10 [*Crassostrea gigas*] | PF00229:TNF(Tumour Necrosis Factor) family |  |
| CL9145.Contig2_All | 1,131 | 2.44 | 4.88 | 3.25 | gi|405970232|gb|EKC35160.1|Tumor necrosis factor ligand superfamily member 10 [*Crassostrea gigas*] | PF00229:TNF(Tumour Necrosis Factor) family |  |
| Unigene45048_All | 1,215 | 7.94 | 6.38 | 3.88 | gi|323134761|gb|ADX31292.1|tumor necrosis factor ligand superfamily member [*Crassostrea gigas*] | PF00229:TNF(Tumour Necrosis Factor) family |  |
| CL1369.Contig1_All | 1,148 | 6.92 | 4.91 | 5.9 | gi|387598305|gb|AFJ91808.1|tumor necrosis factor [*Ostrea edulis*] | PF00229:TNF(Tumour Necrosis Factor) family |  |
| CL1369.Contig2_All | 2,679 | 15.31 | 12.81 | 11.78 | gi|387598305|gb|AFJ91808.1|tumor necrosis factor [*Ostrea edulis*] | PF00229:TNF(Tumour Necrosis Factor) family |  |
| Unigene36334_All | 1,463 | 5.54 | 6.18 | 6.47 | gi|387598305|gb|AFJ91808.1|tumor necrosis factor [*Ostrea edulis*] | PF00229:TNF(Tumour Necrosis Factor) family |  |
| **Lipopolysaccharide-induced tumor necrosis factor-alpha factor** | | | | | |  | Park et al. 2008, *Crassostrea gigas* |
| CL7351.Contig2_All | 500 | 31.43 | 0.56 | 0.31 | gi|405971396|gb|EKC36235.1|Lipopolysaccharide-induced tumor necrosis factor-alpha factor-like protein [*Crassostrea gigas*] | PF10601:LITAF-like zinc ribbon domain |  |
| Unigene45355_All | 538 | 14.14 | 0.83 | 0.48 | gi|405968543|gb|EKC33607.1|Lipopolysaccharide-induced tumor necrosis factor-alpha factor-like protein [*Crassostrea gigas*] | PF10601:LITAF-like zinc ribbon domain |  |
| CL10069.Contig2_All | 653 | 16.64 | 1.11 | 0.16 | gi|282153489|gb|ADA77533.1|lipopolysaccharide-induced TNF-alpha factor [*Meretrix meretrix*] | PF10601:LITAF-like zinc ribbon domain |  |
| CL7351.Contig1_All | 550 | 30.18 | 3.45 | 6.21 | gi|301620244|ref|XP_002939491.1|PREDICTED: lipopolysaccharide-induced tumor necrosis factor-alpha factor homolog isoform 1 [*Xenopus (Silurana) tropicalis*] | PF10601:LITAF-like zinc ribbon domain |  |
| Unigene5253_All | 625 | 10.94 | 3.57 | 2.65 | gi|363902084|gb|AEW43450.1|lipopolysaccharide-induced TNF-alpha factor [*Solen grandis*] | PF10601:LITAF-like zinc ribbon domain |  |
| Unigene37763_All | 655 | 98.92 | 75.98 | 56.64 | gi|405971393|gb|EKC36232.1|Lipopolysaccharide-induced tumor necrosis factor-alpha factor-like protein [*Crassostrea gigas*] | PF10601:LITAF-like zinc ribbon domain |  |
| Unigene20226_All | 778 | 7.37 | 8.82 | 2.86 | gi|405971392|gb|EKC36231.1|Lipopolysaccharide-induced tumor necrosis factor-alpha factor-like protein [*Crassostrea gigas*] | PF10601:LITAF-like zinc ribbon domain |  |
| CL211.Contig1_All | 1,343 | 1.56 | 9.8 | 5.47 | gi|405968535|gb|EKC33599.1|Lipopolysaccharide-induced tumor necrosis factor-alpha factor-like protein [*Crassostrea gigas*] | PF10601:LITAF-like zinc ribbon domain |  |
| **Big defensin (Antimicrobial peptide)** | | |  |  |  |  | Gerdol et al. 2012, *Mytilus galloprovincialis* |
| CL3346.Contig1_All | 512 | 15.62 | 22.34 | 170.79 | gi|74842161|sp|Q86QN6.1|defensin [*Branchiostoma belcheri tsingtauense*] | PF14862:Big defensin |  |
| CL3346.Contig2_All | 222 | 3.23 | 4.02 | 1.86 | gi|332692883|gb|AEE92771.1|big defensin 1 [*Crassostrea gigas*] | PF14862:Big defensin |  |
| CL3346.Contig3_All | 478 | 64.6 | 4.2 | 0.65 | gi|74842161|sp|Q86QN6.1|defensin [*Branchiostoma belcheri tsingtauense*] | PF14862:Big defensin |  |
| **Macin (Antimicrobial peptide)** | |  |  |  |  |  |  |
| Unigene10435_All | 457 | 17.13 | 3.78 | 11.66 | gi|348591863|emb|CCC15019.1|mytimacin-5, partial [*Mytilus galloprovincialis*] | PF14865:Macin |  |
| Unigene46747_All | 374 | 11.06 | 0.01 | 0.01 | gi|348591863|emb|CCC15019.1|mytimacin-5, partial [*Mytilus galloprovincialis*] | PF14865:Macin |  |

#Pfam domain was identified by HMM search against the pfam database, with a evalue threshold of 1e-04.

Table S7: Gene specific primer pairs

|  | Transcript ID no. | Forward primer | Reverse primer |
| --- | --- | --- | --- |
| Gill dominant transcripts | Unigene51974_All | TCCAGGATATGGCACTGTAGGA | TACTGCTGGTATGATTGTTGA |
|  | Unigene36448_All | GGACCTCAGGGATATGCAGGA | TCCAGTTGCTCCTACTTCTC |
|  | CL5231.Contig2_All | ACCAAAGACGAGCAGGAATCA | TCAGCTGGTATTTCACAAGA |
|  | CL79.Contig4_All | CATGGTTGTTGATAACGGA | TCGACTGATGTTCCACCACA |
|  | CL3371.Contig1_All | CATCCGTGTTATGCACGATT | CTTGTTATCAGCACGAGACGT |
|  | CL5089.Contig1_All | AGCAACAATGGGGCAACATCA | AGAATTCGCTTTCGCCGGACA |
|  | CL3148.Contig1_All | CATGCGAAGCACCCGCATGT | ACATGACCTGCTACGTGT |
|  | Unigene43926_All | TCGCCATCATATCTAAATGA | ACAACGATTGTCTAGACAGCACT |
|  | CL5388.Contig1_All | TCTAGGTTACGTTCCACGTACA | TGCCGATGATATTATTCAACA |
|  | Unigene5652_All | CGAACGCATCAAGTACTACGA | TCAATTCTCCCGCTAGCTGT |
| Foot dominant transcripts | CL10676.Contig1_All | GAATACAGCACCTGTTTGTGA | TCTGCATAATTGTCAACCTTGA |
|  | Unigene9278_All | ACAGATTCCTGCAAGCCGAA | TGGCATACAATAACTTAGTATTCAG |
|  | CL9068.Contig1_All | TGCCATGTATTGTAAGTGATTGGT | TCTGGGCATTCTTTTCCACC |
|  | Unigene36314_All | CCAGACACAAGTTGAAGCTGA | GCCATGAGATGTGATAAACTACGT |
|  | Unigene5460_All | ACCCCACAAATTAAACGAAGCA | TGGATTCAGTCTTCTTTTCTTAGT |
|  | CL163.Contig2_All | GTGCACTCCAGTTAGACCAA | ACAGATTCAGAATGATGGTTAGA |
|  | Unigene12641_All | TCAAGCTCCTCTCGTTGGTT | AGAAATAAGAGGCGAAAACA |
|  | CL2829.Contig2_All | CCTCTGATGATTATTAGTCT | TGTCATAGCAGCCGTATAAAAG |
|  | CL3424.Contig1_All | CTTCAACAGATGATGGGACCT | TTCCAGTGTGTATCAGATCTGA |
|  | CL40.Contig5_All | AGTTTGTACCAAGTGCTCTT | CACCATTACGCAGGAAGTGT |
| Mantle dominant transcripts | Unigene24812_All | CCGGTTAATACAATTGTCGTGT | TGCACTAGCGGTGAAATGGT |
|  | CL10404.Contig1_All | AGCTACAGCTTTATGCACTTCCA | CATCCATCTTTCCACTGGGCA |
|  | CL541.Contig3_All | AGTGTGTTTACTGTGGCTACA | TCTGTGTCAGACATTCTGTGT |
|  | CL40.Contig4_All | TGTCTCTGCATTTCTGTAAT | AAACTGGAGCAAGACATTAA |
|  | CL3.Contig28_All | CCTGCCCACGTGTGTCAGT | TCTTTATAAGGTGTGCGCGA |
|  | Unigene39009_All | GATGTGGAGGGAGATAATCT | CAATGATTCAGCTTAAACATGGA |
|  | Unigene63259_All | ACCATAGATCATGTAAAAATCTGT | TGTGCCAATAATCTGAATTCTTGT |
|  | CL7273.Contig1_All | GGGTGGTGACTTGACATGT | GGAACCACCCAATGACCACT |
|  | Unigene13644_All | GTCCTAAATTTTGGCCTGCGT | TGGCGTTGACGCGTTCGAATTGA |
|  | CL11405.Contig1_All | ACATGTCTACAGCCACTGGT | TAATGGGGAGTACGTAGGTGA |
| PGRP | 8290F | gatacccgcggttgggatgat |  |
|  | 8290R | aattcgatcccatcctcgtgc |  |
|  | 2060_1F | AACAAAAAAGGCTGGTCTGAT |  |
|  | 2060_1R | aactctgttccagcctcttcc |  |
|  | 2060_2F | AGTAGACACCGCTGGGATGAT |  |
|  | 2060_2R | aactctgtcccagcctcttcc |  |
|  | 3808F | AACAAAAAACAATTTGACGAC |  |
|  | 3808R | ttctctgtgaaatccccttcc |  |


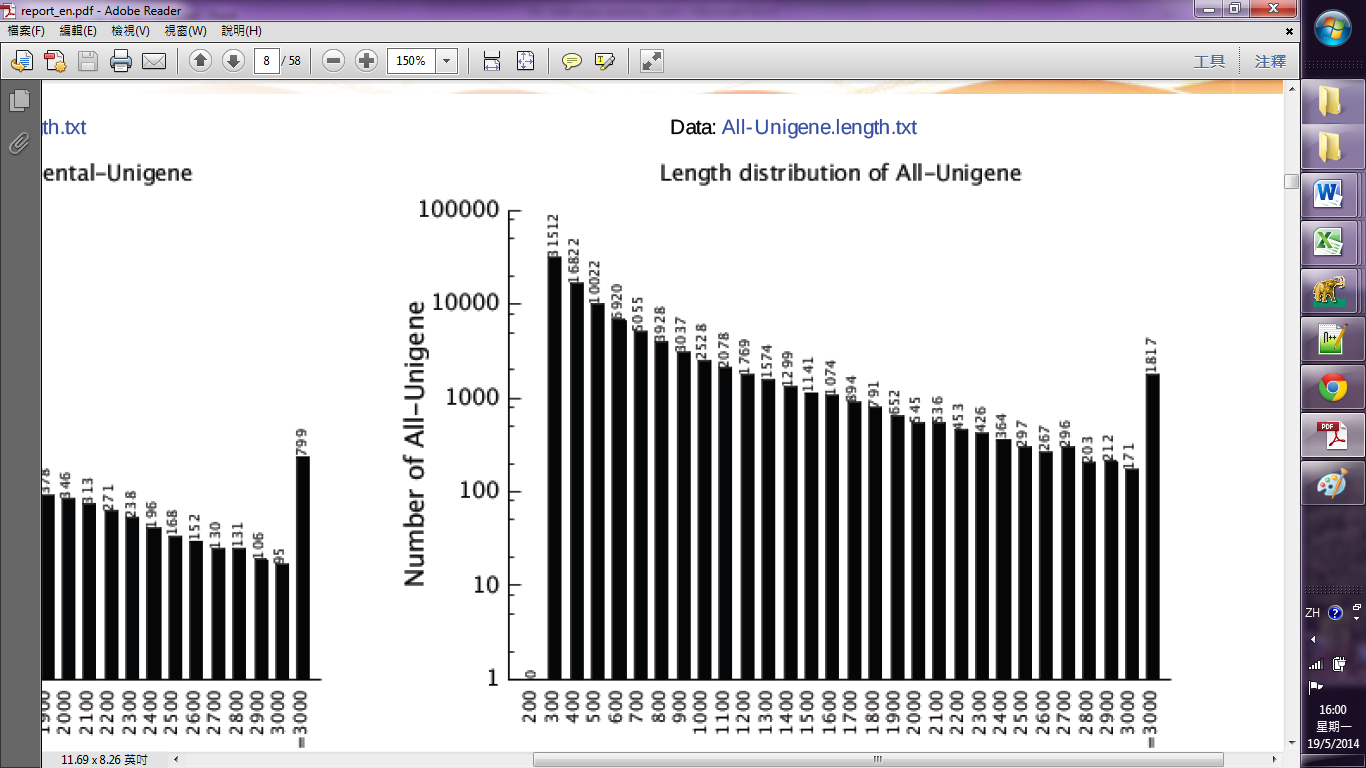


**Figure S1: Length distribution of unigenes in the *B. platifrons* transcriptome**

**
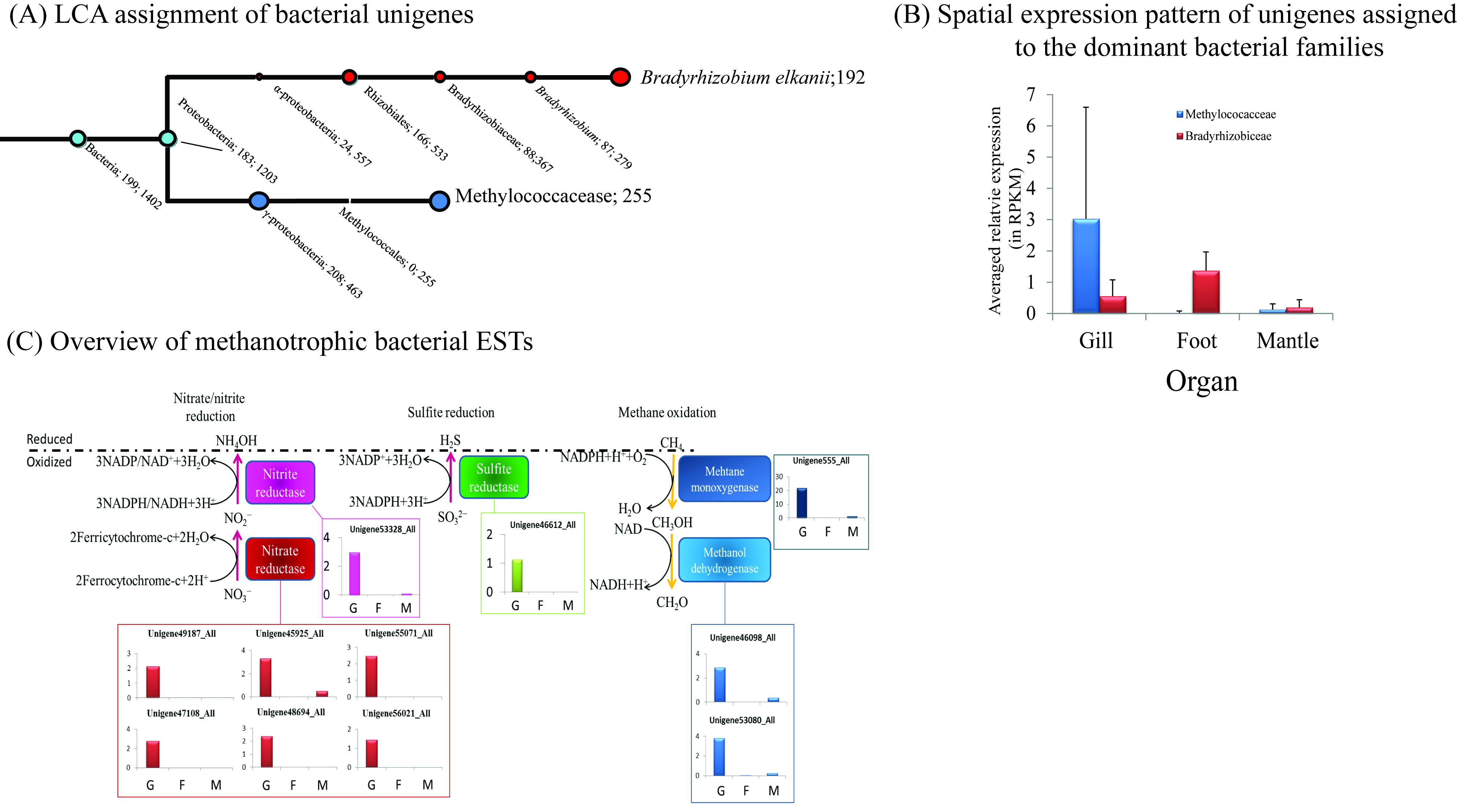
**

**Figure S2. Highlighted metabolic genes transcripts assigned to Methylococcaceae. Note the expression pattern of key enzymes (show in bar-charts) were highly specific to gill.**

**
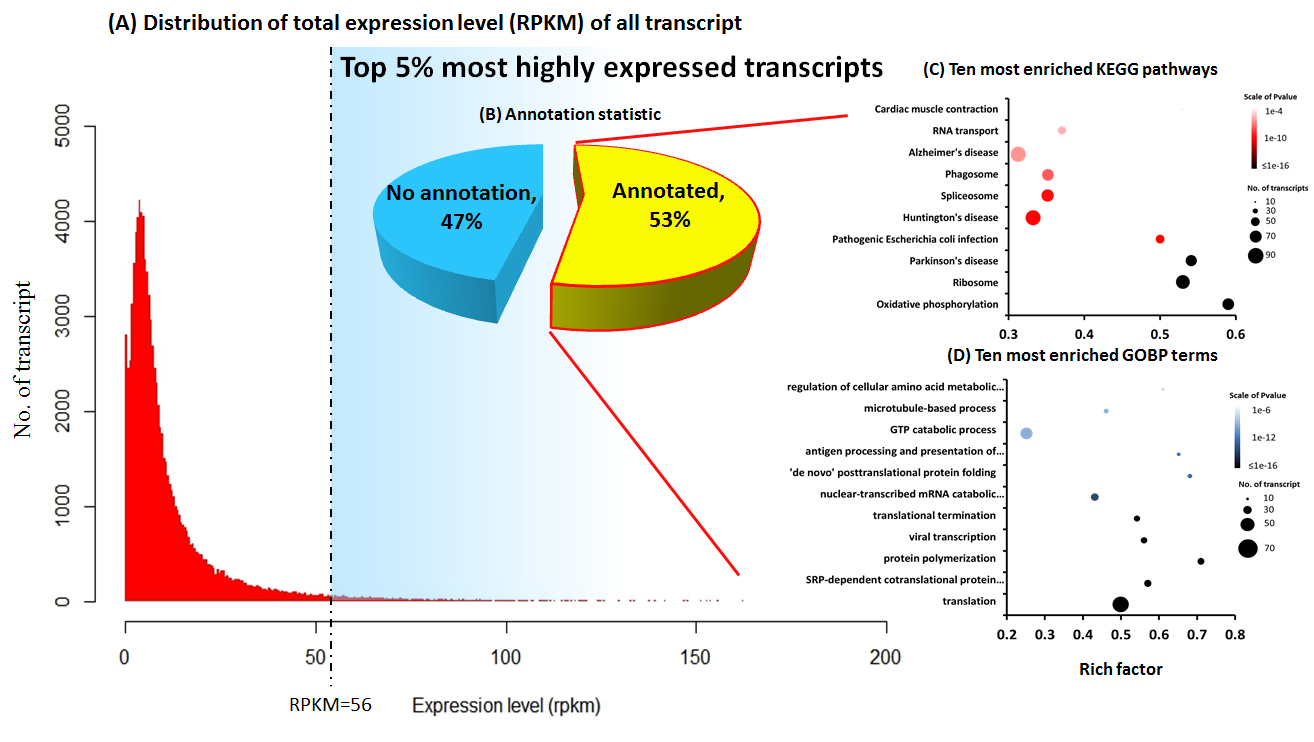
**

**Figure S3. Functional annotation and enrichment analysis of dominant transcripts. (A) Distribution of total expression level (RPKM) of all transcripts in the *B. platifrons* pooled transcriptome. The blue box highlighted the dominant (top5% most highly expressed) transcripts. (B) Annotation overview of the dominant transcripts. (C) The ten most enriched KEGG pathway in the in the top5% most highly expressed transcripts list. (D) The ten most enriched GOBP terms in the top5% most highly expressed transcripts list.**

**
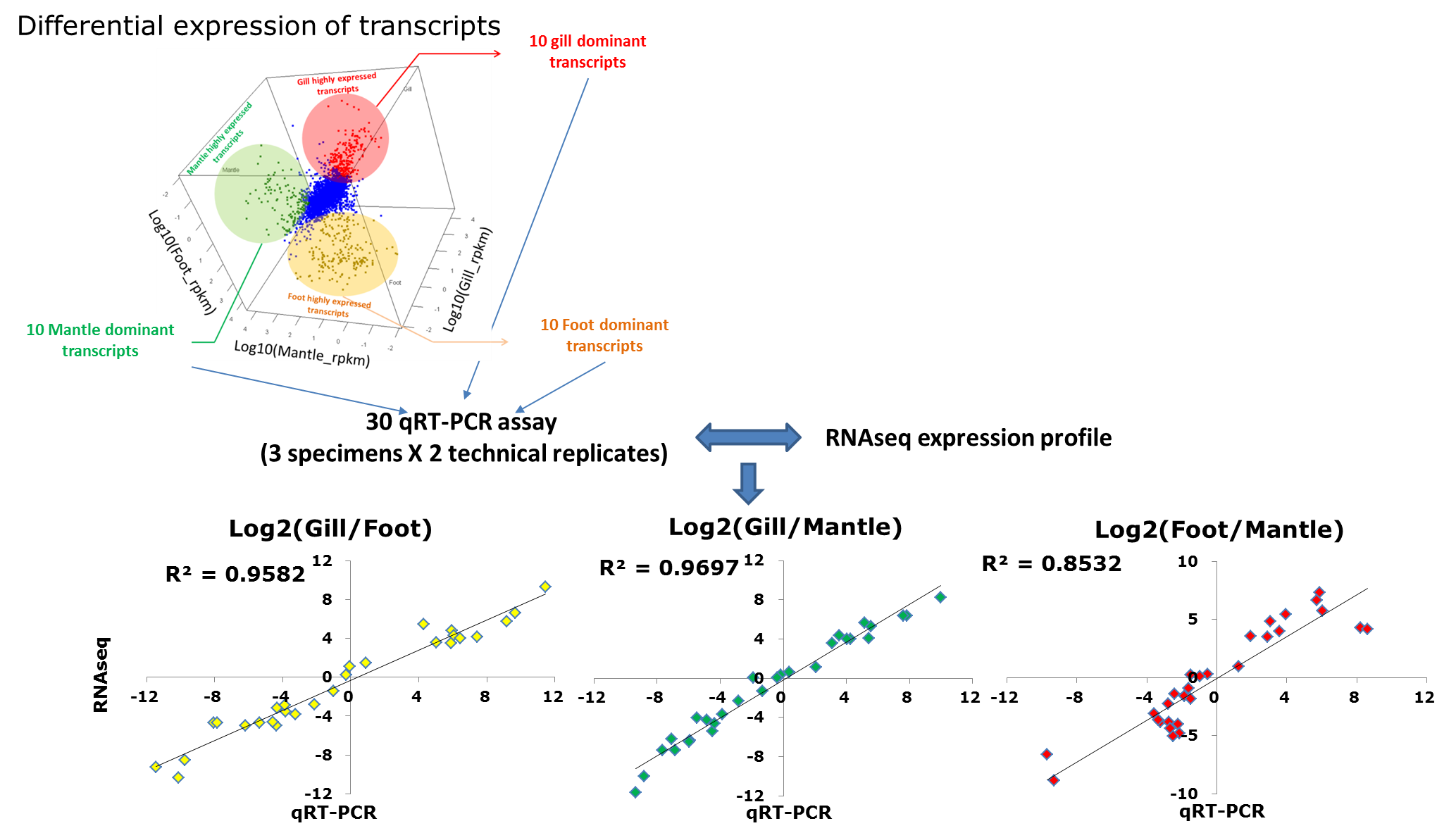
**

**Figure S4. RNAseq quantification versus qRT-PCR assay result. Ten transcripts that displaced differential expression in different examined tissues were randomly selected for qRT-PCR validation. The qRT-PCR results were plot against RNAseq quantification data in three pairwise comparisons: (A) Gill-to-Foot, (B) Gill-to-Mantle and (C) Foot-to-Mantle. The Pearson product-moment correlation coefficients (R^2^) of each plot were shown as an indication of positive linear correlation between the two gene expression quantification methods.**


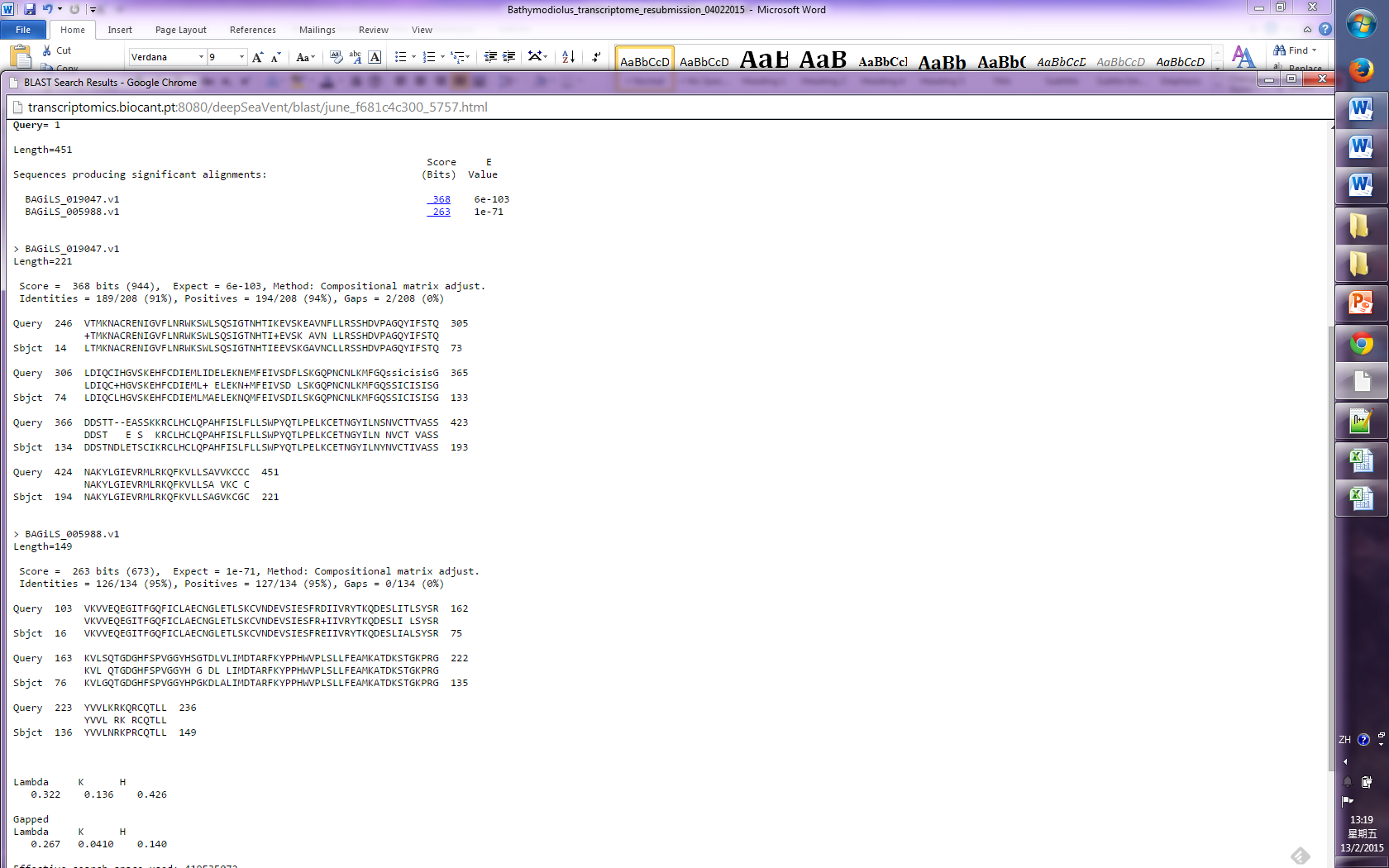


**Figure S5. Blastp search of *B. platifrons* phytochelatin synthase (Unigene479_All) protein sequence against DeepSeaVent database, the customized protein database derived from *B. azoricus* gill transcriptome.**

**
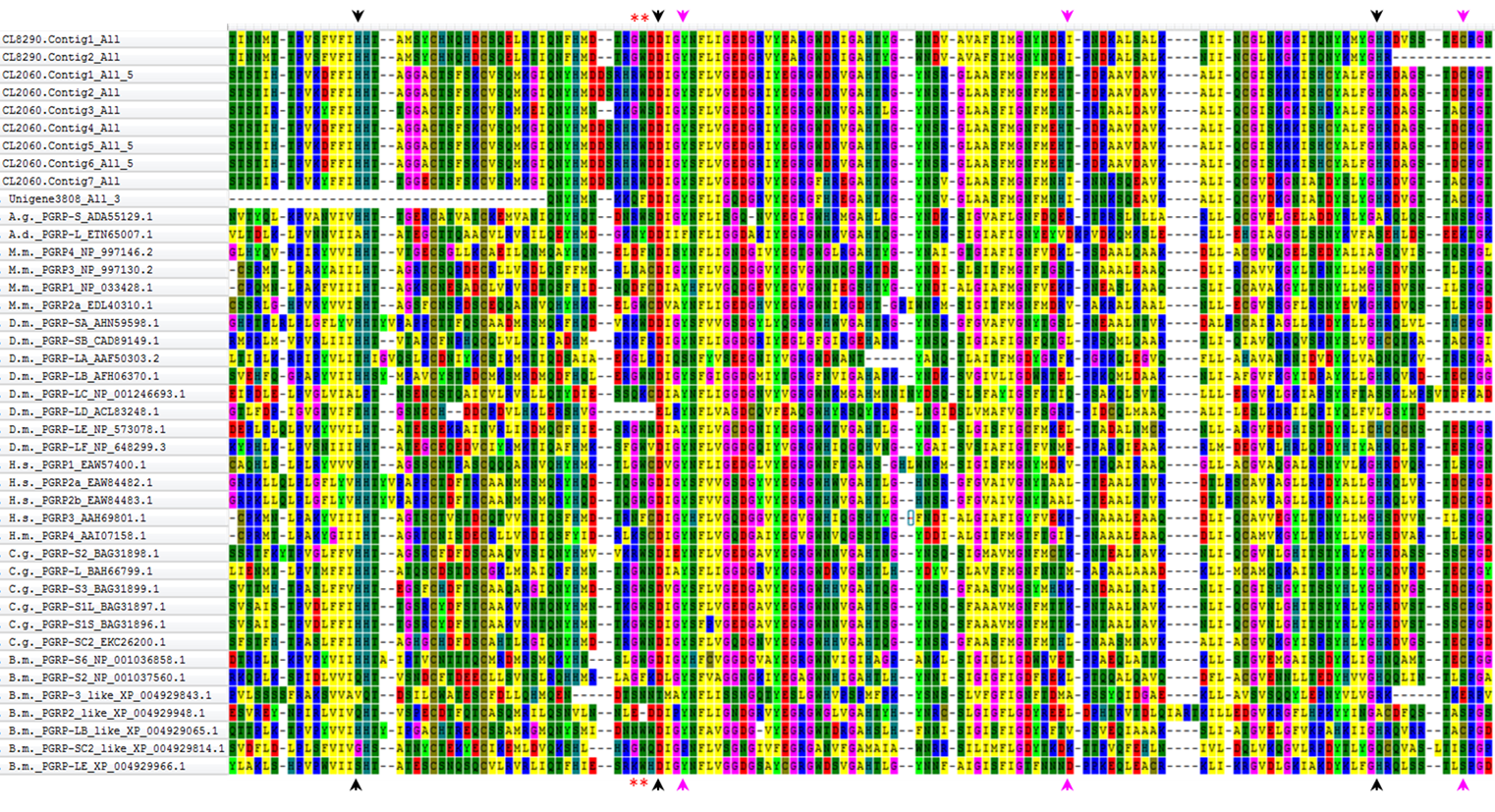
**

**Figure S6. Sequence alignment of amidase domain of *B. platifrons* PGRP against various PGRP splice variants and isoforms from different taxa. Black arrows indicated residues essential for zinc-binding function. Pink arrows indicated residues essential for hydrolytic amidase activity. Red asters indicated the two residues that determine the binding selectivity of PGRPs to Lys-type and Dap-type PGNs.** **A.g.: *Anopheles gambiae*; A.d.: *Anopheles darlingi*; B.m.:*Bombyx mori*; C.g.: *Crassostrea gigas*; D.m: *Drosophila melanogaster*; H.s: *Homo sapiens*; M.m.:*Mus musculus*.**

**Supplemental data file 1.xls:** tblastn result of CEMGA (protein; the query) against *B. platifrons* database (cDNA sequences; the subject). The cutoff value was 1e-05. The first 20 target sequences (at most) is reported.

**Supplemental data file 2.xls:** Expression pattern, taxon and functional annotation of bacterial transcripts in *B. platifrons* transcriptome.
